# Reflection and Transmission Analysis of Surface Acoustic Wave Devices

**DOI:** 10.3390/mi14101898

**Published:** 2023-10-01

**Authors:** Tai-Ho Yu

**Affiliations:** Department of Electronic Engineering, National United University, 2 Lien Da, Nan-Shih Li, Miaoli 36063, Taiwan; yth@nuu.edu.tw

**Keywords:** surface acoustic wave (SAW) filter, wave transfer impedance, reflection and transmittance, constructive interference

## Abstract

This paper presents a study of the propagation of surface acoustic waves in a single and periodic array of metal strip overlays on the surface of layered substrates. Responses of reflected and transmitted surface acoustic waves due to various geometric design parameters of the grating arrays are investigated. An eight-dimensional matrix formulation based on Stroh formalism is adopted to analyze wave propagation in piezoelectric layered media. The dispersion curves for aluminum–zinc oxide films on glass substrates are determined using the surface impedance tensor method. A transfer matrix in terms of the state vectors in cooperation with continuity conditions on the edges of the grating array is used to determine the reflectivity and transmittance of the horizontally propagating surface acoustic waves. The analysis and simulation results show that when the surface acoustic wave is obliquely incident on an array of gratings and the strip width is equal to the gap between strips, the constructive interference of the reflected wave occurs at odd multiples of the strip width to a wavelength ratio of 0.25. When the strip width is unequal to the gap, the constructive interference of the reflected wave is an odd multiple of the strip width to a wavelength ratio of 0.5. An increase in the number of strips concentrates the reflectivity’s extreme frequencies, and an increase in the strip height increases the bandwidth of the extreme frequencies. Both of these increases strengthen the reflected wave’s constructive interferences.

## 1. Introduction

As the technology industry advances, various components are gradually becoming miniaturized, functionally powerful, and precisely actuated. Therefore, piezoelectric materials and their applications are more critical than ever. In 1885, British scholar Rayleigh [1] studied earthquakes and deduced that alongside longitudinal waves and shear waves, a surface acoustic wave (SAW) exists that propagates along the surface of a semi-infinite elastic body. Its polarization direction is located on the sagittal plane. This is spanned by the wave propagation direction and the surface normal vector of the semi-infinite field. Transversely polarized SAWs exist in an isotropic layered structure called Love waves.

In 1965, White et al. [2] developed the interdigitated transducer, which used the piezoelectric properties of quartz substrate to convert electromagnetic waves into mechanical energy and generate Rayleigh waves. This opened the application of SAW components. In 1968 and 1969, Bleustein [3] and Gulyaev [4] discovered that transversely polarized SAWs (SH-SAW) exist in piezoelectric crystals with semi-infinite domains, later called Bleustein–Gulyaev waves. In 1969, Schmidt et al. [5] presented solutions for surface elastic waves propagating in a layered media consisting of a CdS film on a fused quartz substrate. Surface wave velocity, mechanical displacements, and electric fields function as a layer of thickness in Rayleigh and Love modes.

In the design of SAW components with layered structures, Kino et al. [6], in 1973, proposed plating zinc oxide (ZnO) with piezoelectric properties on non-piezoelectric substrates to generate SAWs. In 1977, Shimizu et al. [7] coated zinc oxide on a glass substrate and used interdigitated transducers to produce a SAW element with one-way wave transmission.

In analyzing the wave propagation of SAWs, Braga et al. [8] applied Stroh (1962) [9] to derive the sixth-order matrix of SAWs in the semi-infinite field of anisotropic materials in 1988. They derived the surface wave propagation analysis of anisotropic materials’ layered structures. In 1991, Honein et al. [10] considered electric displacement and potential; they extended the theory to piezoelectric materials and developed an eighth-order piezoelectric wave equation. The concept of the surface impedance tensor proposed by Barnett et al. (1985) [11] was introduced to complete this theoretical derivation.

Tancrell et al. [12] proposed the delta function model in 1971. This model effectively simulates the interdigital transducers’ working frequency. Although it is only suitable for non-dispersive wave propagation and many simplifications have been made, it remains helpful to estimate the frequency response of SAW devices. In 1998, Hachigo et al. [13] made further adjustments to the impulse function model in accordance with the dispersion relationship of wave propagation, increasing the accuracy of the frequency response estimation.

Coupling-of-modes (COM) theory is the most widely used approach to designing and analyzing metal strip arrays. COM theory originated in 1954 and was developed by Pierce [14] during his analysis of optical waveguides. Cross et al. [15] applied this model to the analysis of SAW devices in 1977. Abbott et al. [16,17] made further amendments to the original COM theory in 1989, making it more accurate. COM theory has been widely used in the design and simulation of SAW filters with normal incidence.

In 1999, Royer et al. [18,19] published a book about elastic waves in the solids of piezoelectric material and IDT design. The primary objective of the book was to investigate the methods of generating and detecting bulk and surface elastic waves. It described how their properties could be exploited in constructing various devices.

In 2003, Finger et al. [20] developed and implemented an accurate model for acoustic tracks using 2D approximation only. They used a boundary element formulation to describe piezoelectric substrate’s electro-acoustical properties. This was based on a semi-infinite dyadic Green’s function to avoid compromises in characterizing the physics of bulk–wave interactions. In 2005, Kuypers et al. [21] developed a model to analyze SAW devices based on relating the surface potential adherent to SAWs, propagating on a piezoelectric substrate and applied transducer potential. In 2007, Tagawa [22] proposed an optimum design technique for balanced SAW filters.

In 2012, Tigli et al. [23] presented a finite element modeling and performance analysis of SAW devices developed in complementary metal–oxide–semiconductor (CMOS) technology. In 2013, Elkordy et al. [24] studied the influence of different substrate types, numbers of finger pairs, and finger overlap distances on the unapodized SAW filter’s responses. In 2013, Venkatesan et al. [25] reviewed research on the design and computational modeling of SAW devices and sensors from the previous 20 years. In 2018, Panneerselvan et al. [26] presented a comprehensive review of a SAW sensor’s design, development, simulation, and modeling for potentially sensing toxic gases. In 2019, Wang et al. [27] developed a new approach to analyzing shear horizontal Love wave resonator devices.

In 2021, Shen et al. [28] designed and fabricated wideband SAW filters with series and shunt resonators with different wavelengths and Cu thicknesses. In the same year, Chen et al. [29] proposed a hybrid full-wave analysis of SAW devices to achieve accurate and rapid simulation. Koigerov et al. [30] proposed a numerical approach to extracting COM SAW parameters from periodic structure analyses. COM parameters (velocity, reflection coefficient, effective electromechanical coupling coefficient, and attenuation) were extracted from simulations of test structures using COMSOL Multiphysics. In 2021 and 2023, Su et al. [31,32] proposed a multilayer structure 15YX-LN(300 nm)/SiO_2_(500 nm)/Si to realize wideband SAW filters. They analyzed a wideband SAW filter at 3.7 GHz with spurious mode mitigation using the finite element method.

Various SAW devices have been developed since interdigital transducers (IDTs) were developed. These have been widely used in various fields, especially electronic communication devices. Today, the resonator for signal generation, the sensor for reception, and various filters are common SAW device applications.

The frequency of electronic signals in the communication industry is increasing, and the design and process of the implementation of SAW filters are becoming increasingly challenging. To identify the best design in a limited space, most of the current SAW filters are devoted to developing high frequencies. However, the elastic wave propagation simulation analysis of SAW filters has received limited research attention.

Through the use of continuous wave propagation conditions under different design parameters, it determines the wave transfer impedance of surface acoustic waves in layered media and calculates their dispersion curves using the continuous condition of wave propagation under different design parameters. Moreover, it analyzes the reflection and transmittance changes in SAW’s oblique or vertical incident metal strip arrays to improve the integrity and accuracy of the filter design theory.

## 2. Basic Theorem

### 2.1. Dispersion Equation of SAWs

The governing equations of piezoelectric materials include motion equations and electrostatic equations, which can be expressed as:

(1) $$\sigma_{ij,j}=\rho {\ddot{u}}_{i};\;D_{i,i}=0$$

where $\sigma_{ij}$ is the Cauchy stress tensor, ρ is the mass density of the material, $u_{i}$ is the component of the displacement vector, and $D_{i}$ is the component of the electric displacement vector. The stress component and the electric displacement ϕ are related to the displacement and the electric potential, expressed as the following two formulas, respectively:

(2) $$\sigma_{ij}=C_{ijkl}^{E}u_{k,l}+e_{lij}\phi_{,l};\;D_{i}=e_{ikl}u_{k,l}-\varepsilon_{il}^{S}\phi_{,l}$$

where $C_{ijkl}^{E}$ is the material stiffness coefficient under the fixed electric field strength, $e_{lij}$ and $\varepsilon_{il}^{S}$ are the piezoelectric constant and the dielectric constant under the fixed strain condition, respectively, and the subscript is i,j,k,l=1,2,3.

**Figure 1:**
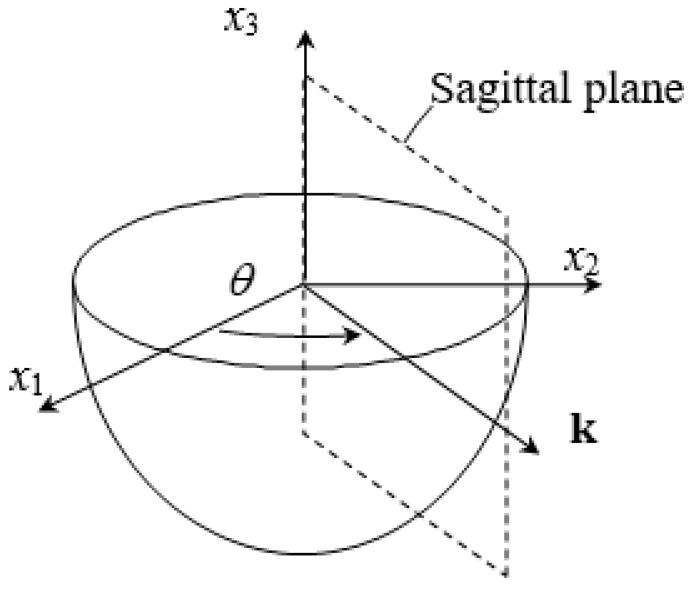
Diagram of material coordinates and wave propagation.

Consider an orthogonal coordinate system and a semi-infinite elastic body, as shown in Figure 1, where $m_{1},m_{2},m_{3}$ are the basis vectors on the three coordinate axes of $x_{1},x_{2},x_{3}$ and the surface normal vector of the r semi-infinite domain is $m_{3}$. The material surface traction vector τ and the electric displacement component $D_{3}$ in the normal direction are, respectively, defined as follows:

(3) $$\tau_{i}=\sigma_{ij}m_{3}=\sigma_{i3};\;D_{3}=D_{i}m_{3}$$

Substitute Equation (2) into Equation (3) and substitute them back to the governing Equation (1) for piezoelectric materials. Extract the state factor of the $x_{3}$-coordinate differential in each formula, respectively, which can be simplified into an eighth-order wave equation [10].

(4) $$\partial_{3}\xi =\Gamma \xi$$

where $\xi ={\left[\begin{array}{cccc} u & iD_{3} & i\tau & \phi \end{array}\right]}^{T}$ is the state vector composed of displacement, electric displacement, traction force, and electric potential.

Define Γ as an 8×8 matrix, and divide Γ into the following sub-matrices:

(5) $$\Gamma =\left[\begin{array}{cccc} \Gamma_{11} & \Gamma_{12} & \Gamma_{13} & \Gamma_{14}\\ \Gamma_{21} & \Gamma_{22} & \Gamma_{23} & \Gamma_{24}\\ \Gamma_{31} & \Gamma_{32} & \Gamma_{33} & \Gamma_{34}\\ \Gamma_{41} & \Gamma_{42} & \Gamma_{43} & \Gamma_{44} \end{array}\right]$$

The derivations of each sub-matrix of Γ are detailed in Appendix A. If a time-harmonic wave propagation along the [cosθ,sinθ,0] directions is considered, the displacement, electric displacement, surface drag, and potential are assumed using the separation of variables method as follows:

(6) $$u(x,x_{3},t)=\bar{u}(x_{3})e^{i(k\cdot x-\omega t)}\;D_{3}(x,x_{3},t)={\bar{D}}_{3}(x_{3})e^{i(k\cdot x-\omega t)}\;\tau (x,x_{3},t)=\bar{\tau }(x_{3})e^{i(k\cdot x-\omega t)}\;\phi (x,x_{3},t)=\bar{\phi }(x_{3})e^{i(k\cdot x-\omega t)}$$

where **k** represents the wave vector, its components in the $x_{1}$ and $x_{2}$ directions are wave numbers $k_{1}$ and $k_{2}$, respectively, and ω is the angular frequency. Assume that the above semi-infinite field is a homogeneous material. If Equation (6) is substituted back into Equation (4), it can be simplified into a differential equation as follows:

(7) $$\partial_{3}\bar{\xi }=iN\bar{\xi };\;\bar{\xi }(x_{3})=e^{iNx_{3}}\bar{\xi }(0)$$

where **N** is an 8×8 material property matrix is unrelated to the *x*_3_ coordinate, and $\bar{\xi }$ is a state vector related to the *x*_3_ coordinate, as follows:

(8) $$\bar{\xi }(x_{3})={\left[\begin{array}{cccc} \bar{u} & i{\bar{D}}_{3} & i\bar{\tau } & \bar{\phi } \end{array}\right]}^{T};\;N=\left[\begin{array}{cccc} N_{11} & N_{12} & N_{13} & N_{14}\\ N_{21} & N_{22} & N_{23} & N_{24}\\ N_{31} & N_{32} & N_{33} & N_{34}\\ N_{41} & N_{42} & N_{43} & N_{44} \end{array}\right]$$

The sub-matrix of the **N** matrix is defined in Appendix A.

Let $\Psi_{\alpha }$ be the eigenvalue of the matrix **N**, ${\left[A_{\alpha },L_{\alpha }\right]}^{T}$ be the corresponding eigenvector, and diagonalize the material matrix **N** as follows:

(9) $$N=\left[\begin{array}{cc} A_{1} & A_{2}\\ L_{1} & L_{2} \end{array}\right]\left[\begin{array}{cc} \Psi_{1} & 0\\ 0 & \Psi_{2} \end{array}\right]{\left[\begin{array}{cc} A_{1} & A_{2}\\ L_{1} & L_{2} \end{array}\right]}^{-1}$$

where $\Psi_{1}=\mathrm{diag}(\zeta_{1},\zeta_{2},\zeta_{3},\zeta_{4})$ and $\Psi_{2}=\mathrm{diag}(\zeta_{5},\zeta_{6},\zeta_{7},\zeta_{8})$. The $\Psi_{\alpha }$ represents the wave number component along the *x*_3_ axis direction.

Subscript 1 represents the harmonic wave propagating or attenuating in the positive *x*_3_ direction (called an up-going wave), and 2 represents the harmonic wave propagating or attenuating in the negative *x*_3_ direction (called a down-going wave). $\zeta_{i}(i=1\sim 8)$ is also referred to as the Floquet wave number of matrix **N**.

By substituting Equation (9) back into Equation (7), $\bar{\xi }(x_{3})$ can be expanded as follows:

(10) $$\bar{\xi }(x_{3})=\left[\begin{array}{cc} A_{1} & A_{2}\\ L_{1} & L_{2} \end{array}\right]\left[\begin{array}{cc} \Phi_{1}(x_{3}) & 0\\ 0 & \Phi_{2}(x_{3}) \end{array}\right]{\left[\begin{array}{cc} A_{1} & A_{2}\\ L_{1} & L_{2} \end{array}\right]}^{-1}\bar{\xi }(0)$$

where $\Phi_{1}(x_{3})={\mathrm{diag}(e}^{i\zeta_{1}x_{3}}{,e}^{i\zeta_{2}x_{3}}{,e}^{i\zeta_{3}x_{3}}{,e}^{i\zeta_{4}x_{3}})$ and $\Phi_{2}(x_{3})={\mathrm{diag}(e}^{i\zeta_{5}x_{3}}{,e}^{i\zeta_{6}x_{3}}{,e}^{i\zeta_{7}x_{3}}{,e}^{i\zeta_{8}x_{3}})$.

The solution of Equation (10) is generally expressed as follows:

(11) $$\left\{\begin{array}{c} \bar{U}(x_{3})\\ \bar{T}(x_{3}) \end{array}\right\}=\left[\begin{array}{cc} A_{1} & A_{2}\\ L_{1} & L_{2} \end{array}\right]\left[\begin{array}{cc} \Phi_{1}(x_{3}) & 0\\ 0 & \Phi_{2}(x_{3}) \end{array}\right]\left\{\begin{array}{c} C_{1}\\ C_{2} \end{array}\right\}$$

where **C_1_** and **C_2_** are constant vectors. The state vectors $\bar{U}(x_{3})$ and $\bar{T}(x_{3})$ are then expressed as follows:

(12) $$\begin{array}{c} \bar{U}(x_{3})={\left[{\bar{u}}_{1}(x_{3}),{\bar{u}}_{2}(x_{3}),{\bar{u}}_{3}(x_{3}),i{\bar{D}}_{3}(x_{3})\right]}^{T}\\ \bar{T}(x_{3})={\left[i{\bar{\tau }}_{1}(x_{3}),i{\bar{\tau }}_{2}(x_{3}),i{\bar{\tau }}_{3}(x_{3}),\bar{\phi }(x_{3})\right]}^{T} \end{array}$$

Define the local impedance $Z_{\alpha }$ as the ratio of the traction force and displacement of a single up- and down-going wave as follows:

(13) $$Z_{\alpha }A_{\alpha }\Phi_{\alpha }C_{\alpha }=L_{\alpha }\Phi_{\alpha }C_{\alpha }$$

or as simplified below:

(14) $$Z_{\alpha }=L_{\alpha }A_{\alpha }{}^{-1}$$

Define the reflection coefficient tensor **R**_0_ as the ratio of the displacement of the up-going wave to the down-going wave at the interface *x_3_* = 0, as follows:

(15) $$A_{1}C_{1}=R_{0}A_{2}C_{2}$$

Substitute Equation (13) and Equation (14) back into Equation (11) to identify the relationship between $\bar{U}(x_{3})$ and $\bar{T}(x_{3})$ as follows:

(16) $$\bar{T}(x_{3})=G(x_{3})\bar{U}(x_{3})$$

(17) $$G(x_{3})=\left[Z_{1}H(x_{3})+Z_{2}\right]{\left[H(x_{3})+I\right]}^{-1}$$

(18) $$H(x_{3})=M_{1}(x_{3})R_{0}M_{2}^{-1}(x_{3})$$

(19) $$M_{\alpha }(x_{3})=A_{\alpha }\Phi_{\alpha }(x_{3})A_{\alpha }^{-1}$$

where $G(x_{3})$ is the wave propagation impedance tensor.

**Figure 2:**
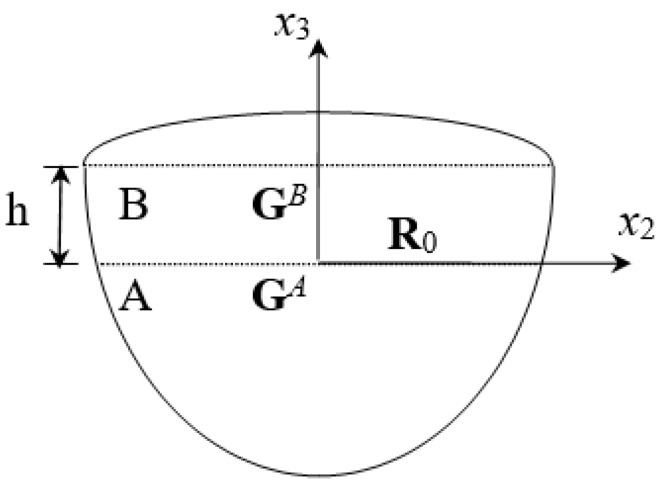
Single-layer semi-infinite domain material.

The superscript represents the substrate’s serial number for the single-layer semi-infinite domain material shown in Figure 2. In base substrate A, because the bottom is a semi-infinite field without up-going waves, meaning that, $A_{1}^{A}\Phi_{1}^{A}=0$, the following equation can be obtained:

(20) $$G^{A}=Z_{2}^{A}$$

In the surface substrate B, assuming that there is a reflecting coefficient tensor **R**_0_ at the boundary with A at *x_3_* = 0, substrate B’s wave propagation impedance can be obtained from Equation (17) as follows:

(21) $$G^{B}(x_{3})=\left[Z_{1}^{B}M_{1}^{B}R_{0}M_{2}^{B}{}^{-1}+Z_{2}^{B}\right]{\left[M_{1}^{B}R_{0}M_{2}^{B}{}^{-1}+I\right]}^{-1}$$

Substrate A and B intersect at *x_3_* = 0, and the condition of boundary continuity must be satisfied; that is, ${\bar{T}}^{A}(0^{+})={\bar{T}}^{B}(0^{-})$ and ${\bar{U}}^{A}(0^{+})={\bar{U}}^{B}(0^{-})$.

Substituting Equations (20) and (21) back into Equation (16), $G^{A}(0^{+})=G^{B}(0^{-})$, yields:

(22) $$R_{0}={\left[Z_{1}^{B}-Z_{2}^{A}\right]}^{-1}\left[Z_{2}^{A}-Z_{2}^{B}\right]$$

**Figure 3:**
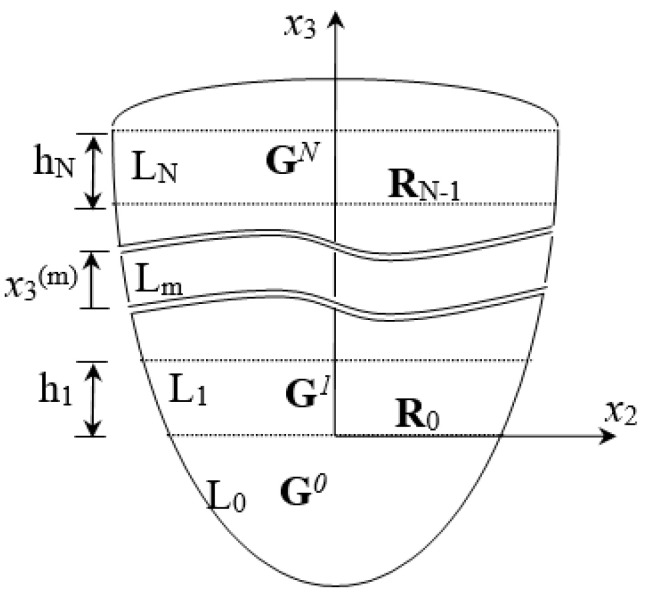
N-layer semi-infinite field materials.

This is extended to N-layer semi-infinite field materials, as shown in Figure 3. L_m_ represents the number of each layer and $x_{3}^{\left(m\right)}=x_{3}-(h_{1}+\ldots +h_{m})>0$. After iterative, the wave impedance and reflection coefficient tensors of each layer can be obtained as follows:

(23) $$\bar{T}(x_{3})=G^{m}(x_{3}^{\left(m\right)})\bar{U}(x_{3})$$

(24) $$G^{0}=Z_{2}^{0};\;G^{m}(x_{3}^{\left(m\right)})=\left[Z_{1}^{m}H^{m}(x_{3}^{\left(m\right)})+Z_{2}^{m}\right]{\left[H^{m}(x_{3}^{\left(m\right)})+I\right]}^{-1}$$

(25) $$H^{m}(x_{3}^{\left(m\right)})=M_{1}^{m}(x_{3}^{\left(m\right)})R_{m-1}M_{2}^{m}{\left(x_{3}^{\left(m\right)}\right)}^{-1};\;M_{\alpha }^{m}(x_{3}^{\left(m\right)})=A_{\alpha }^{m}\Phi_{\alpha }^{m}(x_{3}^{\left(m\right)})A_{\alpha }^{m}{}_{}^{-1}$$

(26) $$R_{m-1}={\left[Z_{1}^{m}-G_{}^{m-1}(h_{m-1})\right]}^{-1}\left[Z_{}^{m-1}(h_{m-1})-Z_{2}^{m}\right];\;Z_{\alpha }^{m}=L_{\alpha }^{m}A_{\alpha }^{m}{}^{-1}$$

where the superscript m represents the substrate L_m_ of thickness *h_m_*, $x_{3}^{\left(m\right)}=x_{3}-h_{\left(m\right)}>0$ and $h_{\left(m\right)}=h_{1}+\ldots +h_{m}$. L_0_ is the base material with a semi-infinite field; **G***^m^* is substrate L_m_‘s impedance tensor. **R**_m_ is the *m*th layer interface’s reflection coefficient tensor, and $Z_{\alpha }^{m}$ is the acoustic wave’s local impedance transmitted up and down the *m*th layer substrate.

When the upper surface of the layer substrate L_N_ is a free boundary, the electric condition circuit is closed (short-circuit), meaning that $\bar{\phi }(h_{\left(N\right)})=0$ and $\bar{T}(h_{\left(N\right)})=0$. Setting $x_{3}=h_{\left(N\right)}$ from Equation (16) yields:

(27) $$G^{N}(h_{N})\bar{U}(h_{\left(N\right)})=0$$

If there is a non-zero solution to $\bar{U}(h)\neq 0$, its sufficient condition is:

(28) $${\left.\det(G^{N})\right|}_{x_{3}^{\left(N\right)}=h_{N}}=0$$

When the upper surface of the layer substrate L_N_ is a free boundary, the electric condition circuit is open (open circuit), meaning that ${\bar{D}}_{3}(h_{\left(N\right)})=0$ and $\bar{T}(h_{\left(N\right)})=[0,0,0,\bar{\phi }]$. Since the potential $\bar{\phi }$ is not limited, the electric displacement and potential-related terms in the wave propagation impedance tensor can be ignored.

Consider Equation (16); if $x_{3}=h_{\left(N\right)}$, this yields:

(29) $${\left.\det(G_{3\times 3}^{N})\right|}_{x_{3}^{\left(N\right)}=h_{N}}=0$$

where $G_{3\times 3}^{N}=\left[\begin{array}{ccc} G_{11}^{N} & G_{12}^{N} & G_{13}^{N}\\ G_{21}^{N} & G_{22}^{N} & G_{23}^{N}\\ G_{31}^{N} & G_{32}^{N} & G_{33}^{N} \end{array}\right]$ is a 3×3 matrix only related to the traction force.

The relationship between k and ω can be obtained from Equation (29); the dispersion equation of the N-layer semi-infinite SAWs.

### 2.2. Rayleigh and Love Waves

Alongside Rayleigh SAWs, SAWs also include transversely polarized horizontal transverse SAWs, also known as Love waves. Therefore, additional restrictions must be added to distinguish these. Figure 1 shows that the propagation directions of the SAWs discussed in this study fall on the quasi-isotropic plane of the layered substrate. The Rayleigh waves only contain the in-plane displacement of the sagittal plane and no out-of-plane displacements. When the wave propagation direction is [cosθ,sinθ,0], its displacement vector can be rewritten as follows:

(30) $$\bar{U}(x_{3})={\left[\bar{u}(x_{3})\cos\theta ,\bar{u}(x_{3})\sin\theta ,{\bar{u}}_{r}(x_{3}),i{\bar{D}}_{3}(x_{3})\right]}^{T}$$

where $\bar{u}(x_{3})$ is the parallel wave vector’s longitudinal displacement, and ${\bar{u}}_{r}(x_{3})$ is the vertical wave vector’s transverse in-plane displacement. If the free boundary ${\bar{\tau }}_{i}(h_{\left(N\right)})=$0 and the electrical boundary condition is closed $\bar{\phi }(h_{\left(N\right)})=0$, the proportional relationship between the displacement components in the $x_{1}$ direction and the $x_{2}$ direction needs to be considered in addition to Equation (28):

(31) $${\left[G{’}_{21}^{N}\cos\theta -G{’}_{11}^{N}\sin\theta \right]}_{x_{3}^{\left(N\right)}=h_{N}}=0$$

where $G{’}_{11}^{N}$ and $G{’}_{21}^{N}$ are elements of the $G{’}_{3\times 1}^{N}=-{\left[\begin{array}{ccc} G_{11}^{N} & G_{12}^{N} & G_{14}^{N}\\ G_{21}^{N} & G_{22}^{N} & G_{24}^{N}\\ G_{41}^{N} & G_{42}^{N} & G_{44}^{N} \end{array}\right]}^{-1}\left[\begin{array}{c} G_{13}^{N}\\ G_{23}^{N}\\ G_{43}^{N} \end{array}\right]$ matrix.

When the electrical boundary condition is open ${\bar{D}}_{3}(h_{\left(N\right)})=0$, the proportional relationship between the $x_{1}$ axis and $x_{2}$ axial displacement components must be considered in addition to Equation (27):

(32) $${\left[\begin{array}{l} (G_{13}^{N}G_{21}^{N}-G_{23}^{N}G_{11}^{N})\cos\theta \\ -(G_{12}^{N}G_{23}^{N}-G_{22}^{N}G_{13}^{N})\sin\theta \end{array}\right]}_{x_{3}^{\left(N\right)}=h_{N}}=0$$

Equations (31) and (32) are the Rayleigh wave dispersion equations for the N-layer semi-infinite field layered substrate when the electrical conditions are closed and open, respectively. Similarly, for the wave propagation of the Love wave, as there is only displacement in the out-of-plane direction, the displacement vector condition can be rewritten as follows:

(33) $$\bar{U}(x_{3})={\left[{\bar{u}}_{s}(x_{3})\sin\theta ,-{\bar{u}}_{s}(x_{3})\cos\theta ,0,i{\bar{D}}_{3}(x_{3})\right]}^{T}$$

where ${\bar{u}}_{s}(x_{3})$ is the displacement in the out-of-plane direction. If there is a free boundary ${\bar{\tau }}_{i}(h_{\left(N\right)})=0$ and the electrical boundary condition is closed $\bar{\phi }(h_{\left(N\right)})=0$, the proportional relationship of the components of the Love wave must be defined in addition to Equation (28):

(34) $${\left[\begin{array}{l} (G_{12}^{N}G_{24}^{N}-G_{22}^{N}G_{14}^{N})\cos\theta \\ +(G_{14}^{N}G_{21}^{N}-G_{24}^{N}G_{11}^{N})\sin\theta \end{array}\right]}_{x_{3}^{\left(N\right)}=h_{N}}=0$$

When the electrical boundary condition is open ${\bar{D}}_{3}(h_{\left(N\right)})=0$, the proportional relationship of displacement must be considered alongside Equation (29):

(35) $${\left[G_{21}^{N}\sin\theta +G_{22}^{N}\cos\theta \right]}_{x_{3}^{\left(N\right)}=h_{N}}=0$$

Therefore, when electrically closed and open, the Love wave dispersion equations for the N-layer semi-infinite field layered substrate are Equation (34) and Equation (35), respectively.

### 2.3. ZnO/Glass and Al/ZnO/Glass SAWs

Zinc oxide has a hexagonal crystal structure (6 mm), and its material has a single axis of symmetry. It is assumed that the axis of symmetry is parallel to the *x*_3_ direction. Due to its high electromechanical coupling coefficient and optical coefficient, zinc oxide is widely used in acoustic waves and optoelectronic devices. Glass is an isotropic material with a slightly lower propagation velocity; therefore, it is unsuitable for making high-frequency devices. However, because of its low price and good light transmittance, it does have advantages in intermediate frequency device applications. In this study, ZnO/Glass and Al/ZnO/Glass layered structures are the main research objects, and SAW phase velocity dispersion curves are calculated for future analysis and discussion.

**Figure 4:**
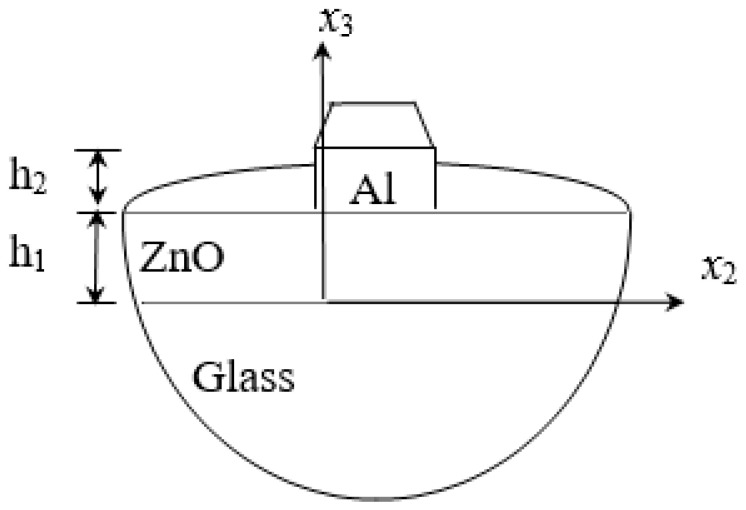
The structure of Al/ZnO/Glass.

In Figure 4, consider that the layered materials are ZnO/Glass and Al/ZnO/Glass. As aluminum (Al) and glass are isotropic materials and the symmetry axis of zinc oxide is perpendicular to the surface of the substrate, the wave propagation of the SAW is not connected to direction. Table 1, Table 2 and Table 3 list the properties of various materials.

**Figure 5:**
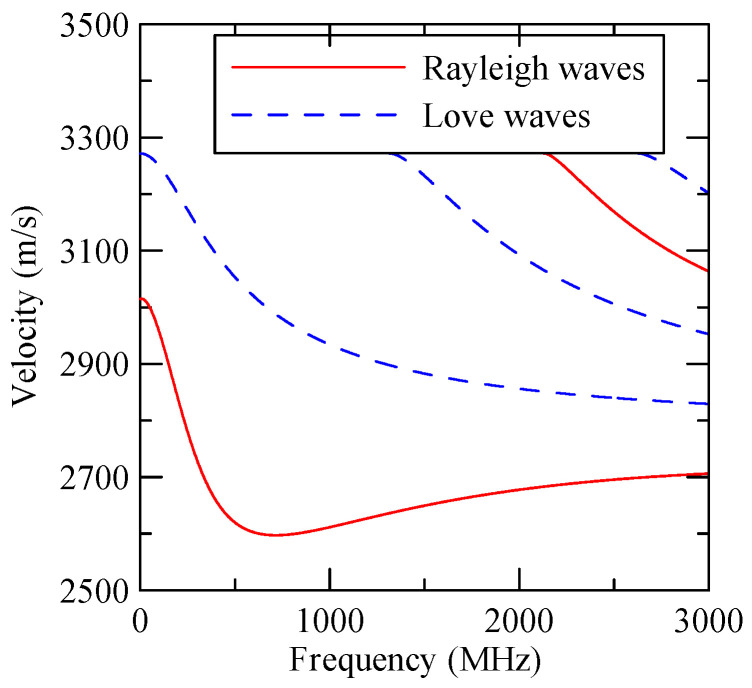
ZnO (1 µm)/glass dispersion curve.

**Figure 6:**
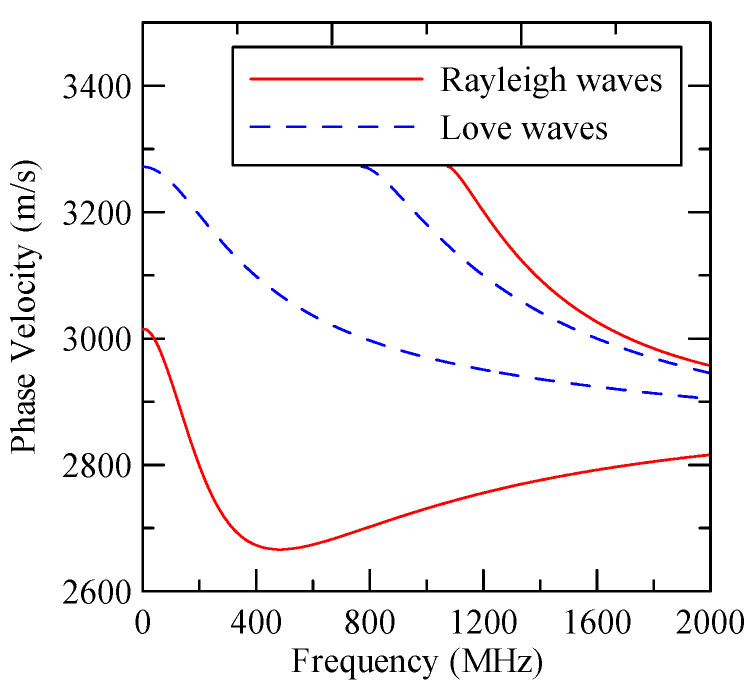
Al (0.5 µm)/ZnO (1 µm)/glass dispersion curve.

**Figure 7:**
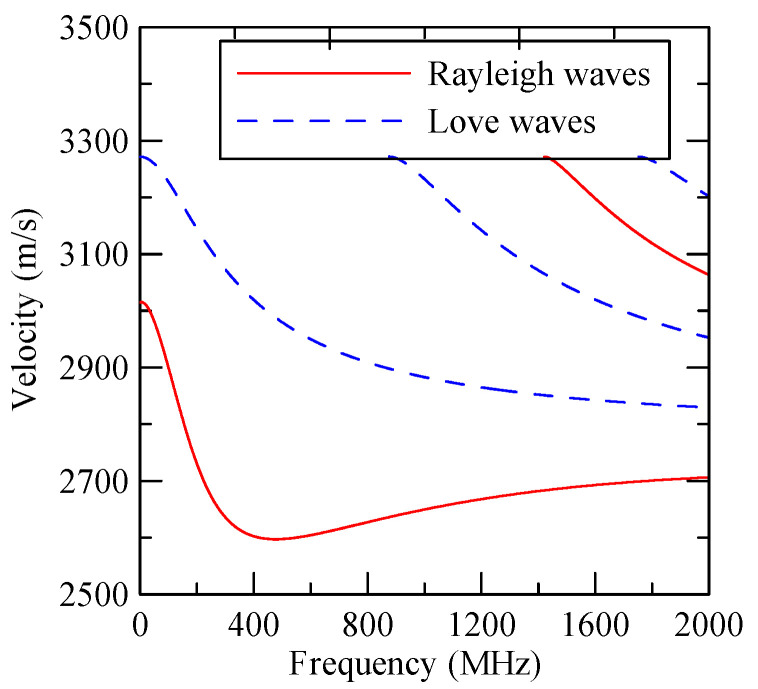
ZnO (1.5 µm)/glass dispersion curve.

**Figure 8:**
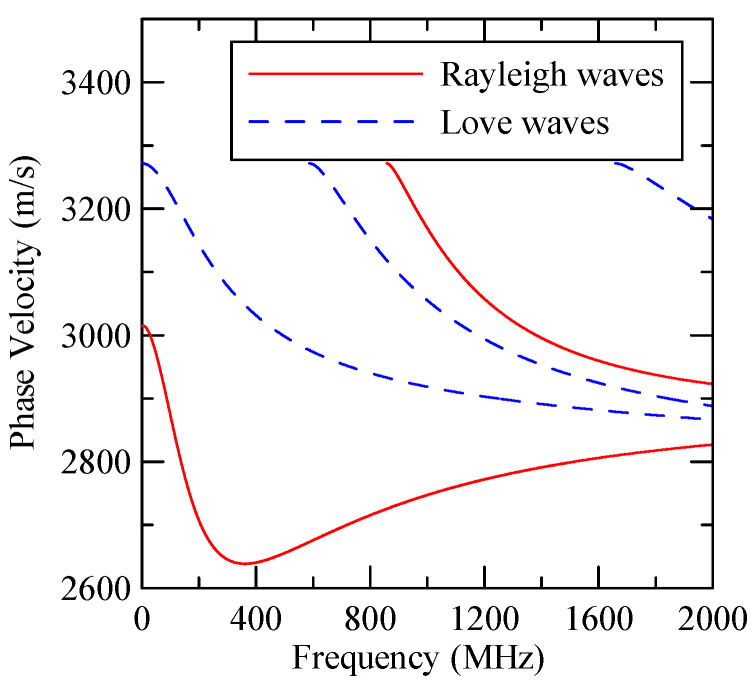
Al (0.5 µm)/ZnO (1.5 µm)/glass dispersion curve.

As the layered semi-infinite field’s upper surface is a free boundary and the electrical boundary is open, the layered substrate is 0.5 mm thick aluminum and 1 mm thick zinc oxide. Furthermore, the thickness of the glass substrate is 1.1 mm. Substituting each parameter into the dispersion relation equation obtains the dispersion curves for ZnO/Glass and Al/ZnO/Glass, which are shown in Figure 5 and Figure 6, respectively. The solid line in the figure represents the Rayleigh wave’s phase velocity, while the dashed line represents the Love wave’s phase velocity. The SAW dispersion curves shown in Figure 7 and Figure 8 demonstrate that the thickness of zinc oxide is 1.5 mm.

As Rayleigh and Love waves only exist at higher frequencies except for the first mode, this study will only discuss the first mode Rayleigh and Love waves.

### 2.4. Delta Function Model

The interdigitated transducer’s delta function model proposed by Tancrell et al. [12,13] estimates the IDT’s frequency response. Although this model makes many simplifications, it is still helpful in the preliminary design of SAW devices.

**Figure 9:**
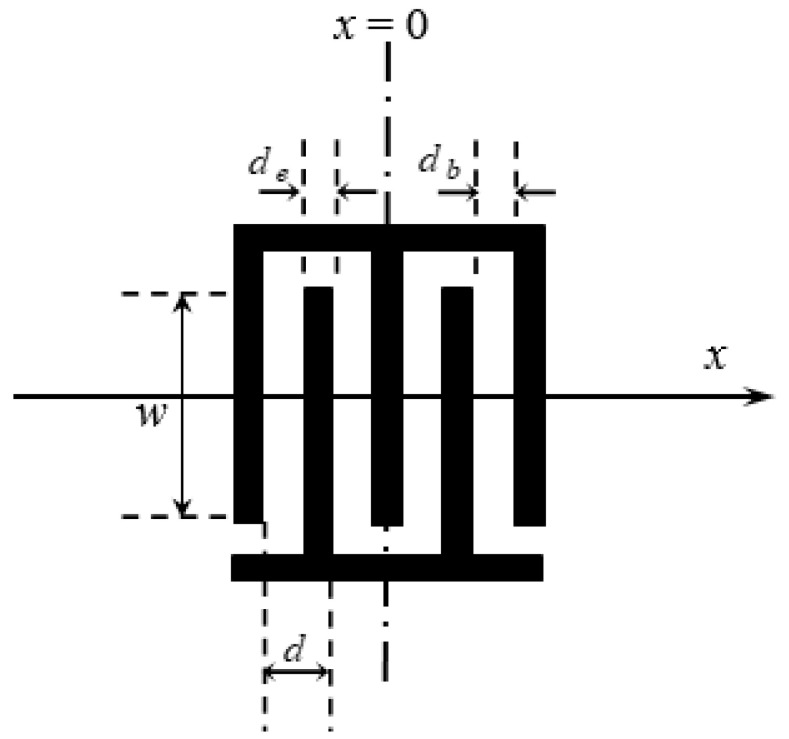
Delta function model of IDT.

As shown in Figure 9, the observation point *x* = 0 is placed in the center of the IDT. Assuming that the electrode cycle is *d*, the pulse generated by the *n*th electrode and its neighboring electrodes can be regarded as the *n*th wave source. $A_{n}$ is its amplitude, which is proportional to the overlapping length w between the electrodes. $s_{n}$ represents the electrode’s polarity, $t_{n}$ is the wave travel time from the *n*th electrode to the observation point, and *N* is the number of electrodes in the IDT. If *N* is an odd number, the observation point falls on the middle electrode; if *N* is an even number, the observation point falls between the two electrodes. Assume that *N* electrodes can generate *N*-1 pulses; then, the overall impulse response of odd electrodes is as follows:

(36) $$h(t)=\sum_{-(N-1)/2}^{(N-1)/2}s_{n}A_{n}\delta (t-t_{n})$$

After Fourier transform, the frequency response can be obtained as:

(37) $$H(f)=\sum_{-(N-1)/2}^{(N-1)/2}s_{n}A_{n}e^{-i2\pi \cdot f\cdot t_{n}}$$

Assuming that the IDT’s pitch is fixed and the overlapping length w between the electrodes is a constant value, if the SAW has no dispersion characteristics, the parameters are defined as follows:

(38) $$\begin{array}{c} s_{n}={\left(-1\right)}^{n};\;d=d_{e}+d_{b}=\mathrm{constant};\;A_{n}=A_{0}=\mathrm{constant};\\ t_{n}=\frac{nd}{v_{R}};\;v_{R}=v_{0}=2df_{0}=\mathrm{constant} \end{array}$$

where $v_{R}=v_{0}$ is the phase velocity of wave propagation, and $f_{0}$ is the designed center frequency. The IDT’s frequency response is:

(39) $$H(f)=A_{0}\sum_{-(N-1)/2}^{(N-1)/2}e^{-in\pi (1+f/f_{0})}$$

Expanding the above equation shows that its imaginary part is an odd function; these cancel out each other. This equation can be simplified as follows:

(40) $$H(f)=A_{0}\sum_{-(N-1)/2}^{(N-1)/2}\cos n\pi \left(1+\frac{f}{f_{0}}\right)$$

The traditional impulse model is discussed for the propagation of non-dispersive waves. To propagate dispersive waves, it must be corrected [15]. If the wave velocity is a function of the working frequency, then Equation (40) can be rewritten as:

(41) $$H(f)=A_{0}\sum_{-(N-1)/2}^{(N-1)/2}\cos n\pi \;\left(\frac{2df}{v_{R}(f)}-1\right);\;t_{n}=\frac{nd}{v_{R}(f)}$$

### 2.5. Al/ZnO/Glass Frequency Response

**Figure 10:**
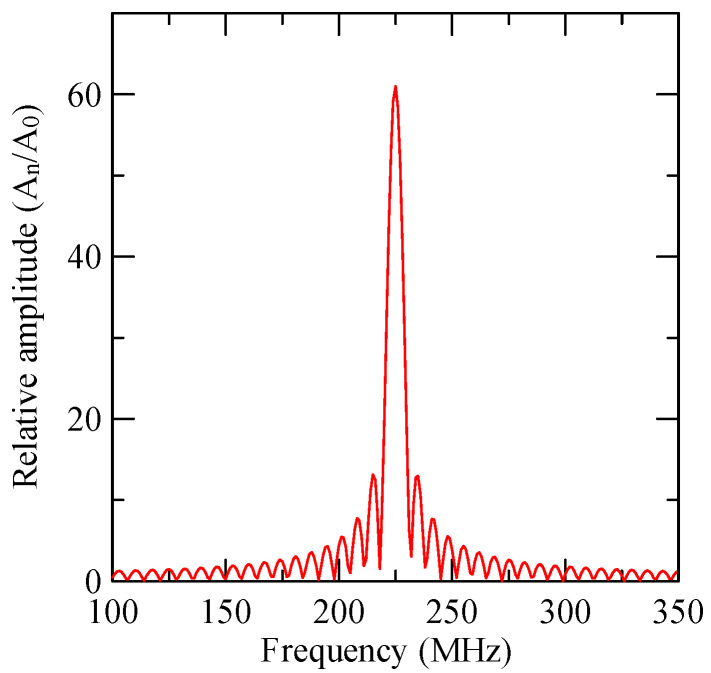
Frequency response of IDT (N = 61, d = 6 μm).

Substituting the first mode Rayleigh wave phase velocity of ZnO (1.5 µm)/glass obtained in the previous section into Equation (41) yields the frequency response shown in Figure 10. Under this design parameter, the Rayleigh SAW device has a maximum response of 224.91 MHz. The Rayleigh wave phase velocity is 2699 m/s, and the Love wave velocity of the same frequency is 3125 m/s.

## 3. Wave Propagation Analysis of Grating Arrays

### 3.1. Horizontal Wave Propagation of SAWs

The dispersion equation’s state vector is simplified using the displacement relationship and boundary conditions. The expression of the SAWs propagating along the surface of the layered substrate can be obtained as follows:

(42) $$\left\{\begin{array}{c} \bar{U}(x_{3})\\ \bar{T}(x_{3}) \end{array}\right\}=\left[\begin{array}{cc} A_{1}^{m} & A_{2}^{m}\\ L_{1}^{m} & L_{2}^{m} \end{array}\right]\left[\begin{array}{cc} \Phi_{1}^{m}(x_{3}^{\left(m\right)}) & 0\\ 0 & \Phi_{2}^{m}(x_{3}^{\left(m\right)}) \end{array}\right]\left\{\begin{array}{c} C_{1}^{m}\\ C_{2}^{m} \end{array}\right\}$$

where $0\leq x_{3}^{\left(m\right)}\leq h_{m}$, $\bar{U}(x_{3})$, and $\bar{T}(x_{3})$ are state vectors. The undetermined coefficient vector C can be expressed as follows:

(43) $$\left[\begin{array}{c} C_{1}^{m}\\ C_{2}^{m} \end{array}\right]={\left[\begin{array}{cc} A_{1}^{m} & A_{2}^{m}\\ L_{1}^{m} & L_{2}^{m} \end{array}\right]}^{-1}\;\sum_{m}^{N}\left(\left[\begin{array}{cc} A_{1}^{m} & A_{2}^{m}\\ L_{1}^{m} & L_{2}^{m} \end{array}\right]\;\Pi^{m}\right)\;\bar{Κ}\;u$$

(44) $$\Pi^{m}={\left[\begin{array}{cc} \Phi_{1}^{m}(h_{m}) & 0\\ 0 & \Phi_{2}^{m}(h_{m}) \end{array}\right]}^{-1}{\left[\begin{array}{cc} A_{1}^{m} & A_{2}^{m}\\ L_{1}^{m} & L_{2}^{m} \end{array}\right]}^{-1}$$

where u is the amplitude of the surface displacement of the substrate and $\bar{K}$ is the state vector of the layered surface. The derivation of this is detailed in Appendix B. When the wave propagates as a Rayleigh wave, $\bar{Κ}={\left[\begin{array}{cccccccc} a_{0}\cos\theta & a_{0}\sin\theta & 1 & a_{1} & 0 & 0 & 0 & a_{2} \end{array}\right]}^{T}$.

If the electrical boundary condition is open ${\bar{D}}_{3}(h_{\left(N\right)})=0$, this yields:

(45) $$\begin{array}{c} a_{0}=\left\{\begin{array}{c} {\left[\frac{G_{23}^{N}G_{31}^{N}-G_{21}^{N}G_{33}^{N}}{G_{21}^{N}G_{32}^{N}-G_{22}^{N}G_{31}^{N}}\csc\theta \right]}_{x_{3}^{\left(N\right)}=h_{N}},\;if\;\theta \neq n\pi \\ {\left[\frac{G_{12}^{N}G_{23}^{N}-G_{22}^{N}G_{13}^{N}}{G_{11}^{N}G_{22}^{N}-G_{21}^{N}G_{12}^{N}}\sec\theta \right]}_{x_{3}^{\left(N\right)}=h_{N}},if\;\theta \neq (n-0.5)\pi \end{array}\right.;\\ a_{1}=0;\;a_{2}={\left[a_{0}(G_{41}^{N}\cos\theta +G_{42}^{N}\sin\theta )+G_{43}^{N}\right]}_{x_{3}^{\left(N\right)}=h_{N}} \end{array}$$

When the electrical boundary condition is closed $\bar{\phi }(h_{\left(N\right)})=0$, then:

(46) $$\begin{array}{c} a_{0}=\left\{\begin{array}{c} {\left[G{’}_{21}^{N}\csc\theta \right]}_{x_{3}^{\left(N\right)}=h_{N}},\;if\;\theta \neq n\pi \\ {\left[G{’}_{11}^{N}\sec\theta \right]}_{x_{3}^{\left(N\right)}=h_{N}},if\;\theta \neq (n-0.5)\pi \end{array}\right.;\\ a_{1}={\left(G{’}_{31}^{N}\right)}_{x_{3}^{\left(N\right)}=h_{N}};\;a_{2}={\left[a_{0}(G_{41}^{N}\cos\theta +G_{42}^{N}\sin\theta )+G_{43}^{N}+a_{1}G_{44}^{N}\right]}_{x_{3}^{\left(N\right)}=h_{N}} \end{array}$$

where $G{’}^{N}$ is defined in Equation (31). Similarly, when the wave propagates as a Love wave, the state vector of the layered substrate surface can be expressed as $\bar{Κ}={\left[\begin{array}{cccccccc} \sin\theta & -\cos\theta & 0 & b_{0} & 0 & 0 & 0 & b_{1} \end{array}\right]}^{T}$.

When the electrical boundary condition is open ${\bar{D}}_{3}(h_{\left(N\right)})=0$, then:

(47) $$b_{0}=0;\;b_{1}={\left[G_{41}^{N}\sin\theta -G_{42}^{N}\cos\theta \right]}_{x_{3}^{\left(N\right)}=h_{N}}$$

When the electrical boundary condition is closed $\bar{\phi }(h_{\left(N\right)})=0$, then:

(48) $$\begin{array}{c} b_{0}=\left\{\begin{array}{c} {\left(\frac{G_{11}^{N}G_{42}^{N}-G_{41}^{N}G_{12}^{N}}{G_{12}^{N}G_{44}^{N}-G_{42}^{N}G_{14}^{N}}\sin\theta \right)}_{x_{3}^{\left(N\right)}=h_{N}}\;,\;if\;\theta \neq n\pi \\ {\left(\frac{G_{21}^{N}G_{44}^{N}-G_{41}^{N}G_{24}^{N}}{G_{12}^{N}G_{44}^{N}-G_{42}^{N}G_{14}^{N}}\cos\theta \right)}_{x_{3}^{\left(N\right)}=h_{N}},\;if\;\theta \neq (n-0.5)\pi \end{array}\right.\\ b_{1}={\left(G_{41}^{N}\sin\theta -G_{42}^{N}\cos\theta +b_{0}G_{44}^{N}\right)}_{x_{3}^{\left(N\right)}=h_{N}} \end{array}$$

### 3.2. Reflection and Transmission on Grating Arrays

If the substrate’s shape or material changes, reflection and transmission wave propagation will occur on the SAW transmission path traveling along the substrate’s surface. In this study, the reflection and transmittance of the array strip’s SAWs are deduced relative to the substrate change caused by the strip array. Most scholars have previously used the reflection coefficient and transmission coefficient of SAWs as the unknown variables. Conversely, this study uses the state vector of SAWs as the unknown variable. The wave propagation continuity condition on both sides of the strip array is used to solve the problem, and the ratio of the state vector’s corresponding components is calculated to obtain the reflectivity and transmittance.

**Figure 11:**
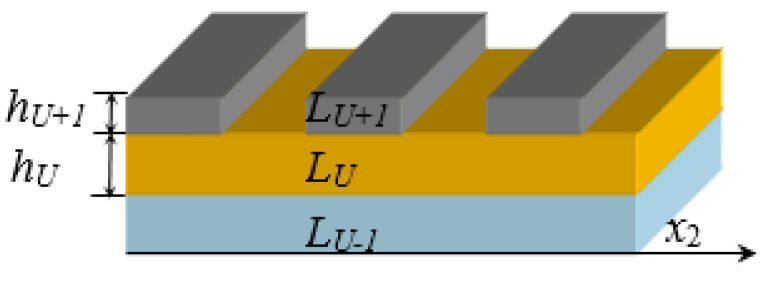
Cross-sectional view of reflective strip array.

**Figure 12:**
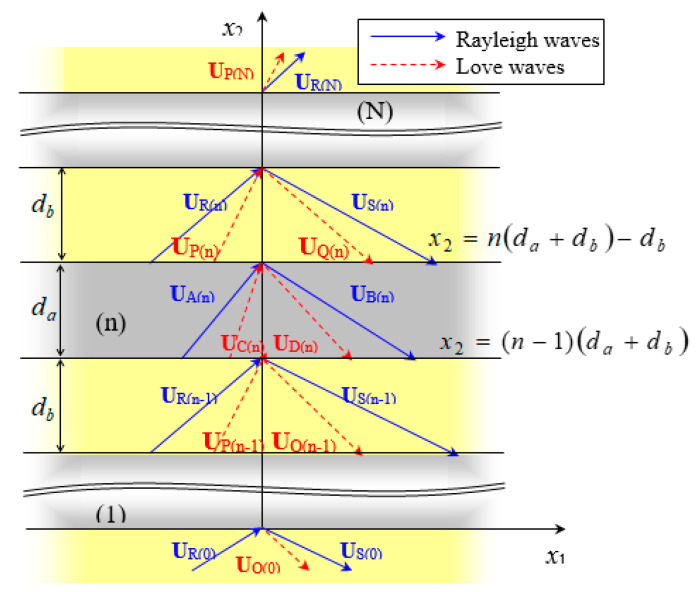
Diagram of strip array and wave propagation.

Consider a Rayleigh wave $U_{R(0)}$ with propagation direction $N_{R}$ passing through the strip array shown in Figure 11 and Figure 12. $L_{U}$ shown in Figure 11 is the surface medium, and $L_{U+1}$ is the strip array layer. $U_{H}$ in Figure 12 is the reflection and transmission wave propagation, and $N_{H}$ is the wave propagation direction. The subscripts H=A~D and P~S represent wave propagation in the strip and non-strip regions, respectively, where R,S,A,B are Rayleigh waves, P,Q,C,D are Love waves, and $d_{a}$ and $d_{b}$ represent the width and spacing of strips, respectively.

If the electrical boundary condition on the upper surface of the strip array (the $x_{3}$ direction) is open, this can be obtained as follows:

(49) $$\begin{array}{cc} \left\{\begin{array}{c} U_{H}(x,x_{3})e^{-i\omega t}\\ D_{3H}e^{-i\omega t} \end{array}\right\} & ={\bar{U}}_{H}(x_{3})e^{i(k_{H}N_{H}\cdot x-\omega t)}\\ & =\left\{\begin{array}{c} H_{3\times 1}(x_{2},x_{3})\\ 0 \end{array}\right\}u_{H}e^{i(k_{H}\cos\theta_{H}x_{1}-\omega t)} \end{array}$$

(50) $$\begin{array}{cc} \sigma_{23}^{\left(H\right)}(x,x_{3})e^{-i\omega t} & ={\bar{\tau }}_{2H}^{}(x_{3})e^{i(k_{H}N_{H}x-\omega t)}\\ & =\tau_{2H}^{}(x_{2},x_{3})u_{H}e^{i(k_{H}\cos\theta_{H}x_{1}-\omega t)} \end{array}$$

Here, the vector **H** = **A**, **B**, **R**, **S** represents the Rayleigh wave’s displacement vector. If the vector **H** = **C**, **D**, **P**, **Q**, this represents the displacement vector of the Love wave. Considering the phenomenon of dispersion, wave number $k_{H}=$ $\omega /v_{H}$, where the wave velocity $v_{H}=v_{H}(\omega )$ is a frequency function.

When a time-harmonic SAW passes through the strip array, the wave propagation displacement below the upper surface of the layered medium ($x_{3}\leq h_{\left(U\right)}$) is continuous. Furthermore, the displacement vector passing through the front and rear edges of the *n*th strip is:

(51) $$\underset{H=R,S,P,Q}{\sum }\left(U_{H(n-1)}(x_{n-1},x_{3})\right)=\sum_{H=A,B,C,D}\left(U_{H(n)}(x_{n-1},x_{3})\right)\;\sum_{H=A,B,C,D}\left(U_{H(n)}(x_{n},x_{3})\right)=\sum_{H=R,S,P,Q}\left(U_{H(n)}(x_{n},x_{3})\right)$$

where the position vectors are $x_{n-1}=[x_{1},(n-1)(d_{a}+d_{b})]$ and $x_{n}=[x_{1},n(d_{a}+d_{b})-d_{b}]$.

Alongside continuous displacement, the stress component $\sum \sigma_{2i}^{\left(H\right)}$ in the $x_{2}$ direction is also continuous in the area ($x_{3}\leq h_{\left(U\right)}$) below the strip array’s surface:

(52) $$\sum_{H=R,S,P,Q}\left(\sigma_{23}^{H(n-1)}(x_{n-1},x_{3})\right)=\sum_{H=A,B,C,D}\left(\sigma_{23}^{H(n)}(x_{n-1},x_{3})\right)\;\sum_{H=A,B,C,D}\left(\sigma_{23}^{H(n)}(x_{n},x_{3})\right)=\sum_{H=R,S,P,Q}\left(\sigma_{23}^{H(n)}(x_{n},x_{3})\right)$$

If the SAW is a plane wave, the array strips are infinitely long in the *x*_1_ direction. As the continuous condition of wave propagation is not affected by the parameters in the $x_{1}$ direction, the wave propagation factor in the $x_{1}$ direction can be extracted to obtain an identity as follows:

(53) $$e^{ik_{H}x_{1}\cos\theta_{H}}=e^{k_{R}x_{1}\cos\theta_{R}}$$

As the $x_{1}$ coordinate is an arbitrary value, the wave numbers of the reflected and transmitted waves in the $x_{1}$ direction are equal:

(54) $$k_{H}\cos\theta_{H}=k_{R}\cos\theta_{R}$$

This is the so-called Snell’s Law, as follows:

(55) $$k_{H}\sin\Theta_{H}=k_{R}\sin\Theta_{R}$$

where $\Theta_{R}=\pi /2-\theta_{R}$ represents the incident angle, and $\Theta_{H}=\pi /2-\theta_{H}$ represents the reflection and transmission angles of each mode-converted SAW.

When the wave propagates through the *n*th strip, its displacement change is expressed as follows:

(56) $$u_{\left(n\right)}=M_{\left(n\right)}u_{\left(n-1\right)}$$

where $u_{\left(n\right)}$ is the displacement state vector passing through the *n*th fringe, and $M_{\left(n\right)}$ is a 4×4 wave propagation matrix. They are, respectively, expressed as follows:

(57) $$u_{\left(n\right)}={\left[\begin{array}{cccc} u_{R(n)} & u_{S(n)} & u_{P(n)} & u_{Q(n)} \end{array}\right]}^{T}$$

(58) $$M_{\left(n\right)}(x_{3})={\left[M_{1}^{-1}M_{2}\right]}_{x_{2}=x_{2(n)}}{\left[M_{2}^{-1}M_{1}\right]}_{x_{2}=x_{2(n-1)}}$$

(59) $$M_{1}(x_{2},x_{3})=\left[\begin{array}{cccc} R_{3\times 1} & S_{3\times 1} & P_{3\times 1} & Q_{3\times 1}\\ \tau_{2R} & \tau_{2S} & \tau_{2P} & \tau_{2Q} \end{array}\right];\;M_{2}(x_{2},x_{3})=\left[\begin{array}{cccc} A_{3\times 1} & B_{3\times 1} & C_{3\times 1} & D_{3\times 1}\\ \tau_{2A} & \tau_{2B} & \tau_{2C} & \tau_{2D} \end{array}\right]$$

Here, $x_{2(n-1)}=(n-1)(d_{a}+d_{b})$ and $x_{2(n)}=n(d_{a}+d_{b})-d_{b}$. When the Rayleigh wave is vertically incident on the strips (i.e., $\Theta_{R}=0$), only the Rayleigh wave remains in the reflected and transmitted waves. The Love wave will no longer exist. Therefore, Equation (56) can be simplified as follows:

(60) $$\left\{\begin{array}{c} u_{R(n)}\\ u_{S(n)} \end{array}\right\}={\bar{M}}_{\left(n\right)}\left\{\begin{array}{c} u_{R(n-1)}\\ u_{S(n-1)} \end{array}\right\}$$

where ${\bar{M}}_{\left(n\right)}$ is a 2×2 wave propagation matrix, then:

(61) $${\bar{M}}_{\left(n\right)}(x_{3})={\left[{\bar{M}}_{1}^{-1}{\bar{M}}_{2}\right]}_{x_{2}=x_{2(n)}}{\left[{\bar{M}}_{2}^{-1}{\bar{M}}_{1}\right]}_{x_{2}=x_{2(n-1)}}$$

(62) $${\bar{M}}_{1}(x_{2},x_{3})=\left[\begin{array}{cc} R_{2} & S_{2}\\ R_{3} & S_{3} \end{array}\right];\;{\bar{M}}_{2}(x_{2},x_{3})=\left[\begin{array}{cc} A_{2} & B_{2}\\ A_{3} & B_{3} \end{array}\right]$$

where $R_{i},S_{i},A_{i},B_{i}$ are the elements of $R_{3\times 1},S_{3\times 1},A_{3\times 1},B_{3\times 1}$, respectively. Extending the above derivation to *N* strips array, the state equation of the global SAW can be obtained as follows:

(63) $$u_{\left(N\right)}=\sum_{n=N}^{1}\left(M_{\left(n\right)}\right)u_{\left(0\right)},\;\Theta_{R}>0$$

(64) $$\left\{\begin{array}{c} u_{R(N)}\\ u_{S(N)} \end{array}\right\}=\sum_{n=N}^{1}\left({\bar{M}}_{\left(n\right)}\right)\left\{\begin{array}{c} u_{R(0)}\\ u_{S(0)} \end{array}\right\},\;\Theta_{R}=0$$

Calculating the ratio of the displacements $u_{S(0)}$ and $u_{R(0)}$ of the reflected and transmitted fields to the displacement $u_{R(0)}$ of the incident Rayleigh wave obtains the reflection and transmittance of the Rayleigh wave passing through the array strip. The transmission and reflectivity of Rayleigh waves are defined as follows:

(65) $$\left|T_{rr}\right|={\left|\frac{u_{R(N)}}{u_{R(0)}}\right|}^{2};\;\left|R_{rr}\right|={\left|\frac{u_{S(0)}}{u_{R(0)}}\right|}^{2}$$

**Figure 13:**
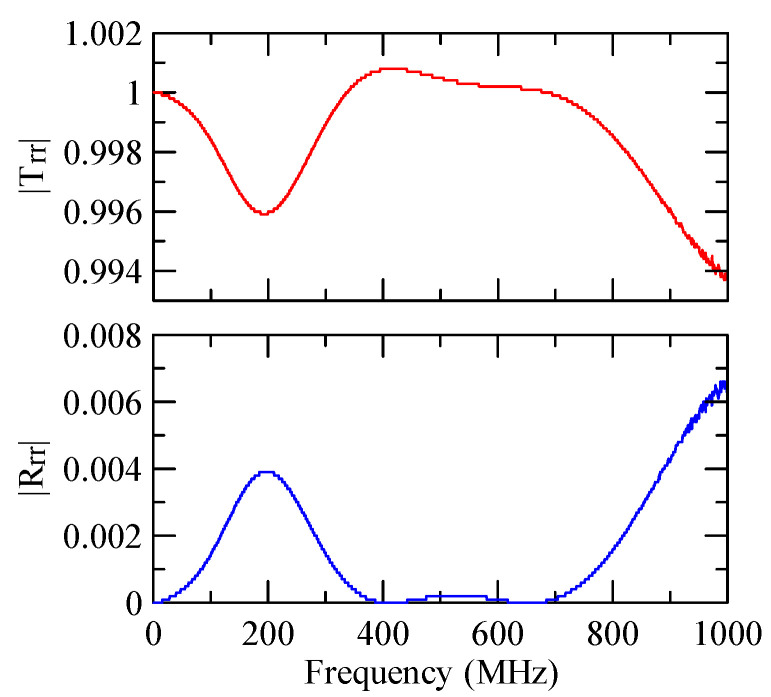
Reflection and transmission spectrum of $d_{a}=3\;\mu m$, $\Theta_{R}=45{}^{\circ}$, N = 1.

**Figure 14:**
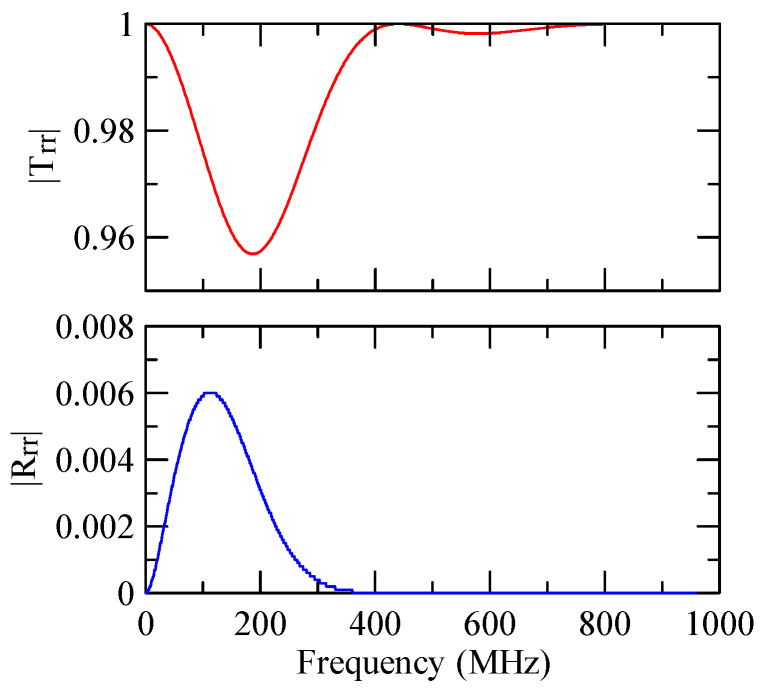
Reflection and transmission spectrum of $d_{a}=3\;\mu m$, $\Theta_{R}=0{}^{\circ}$, N = 1.

**Figure 15:**
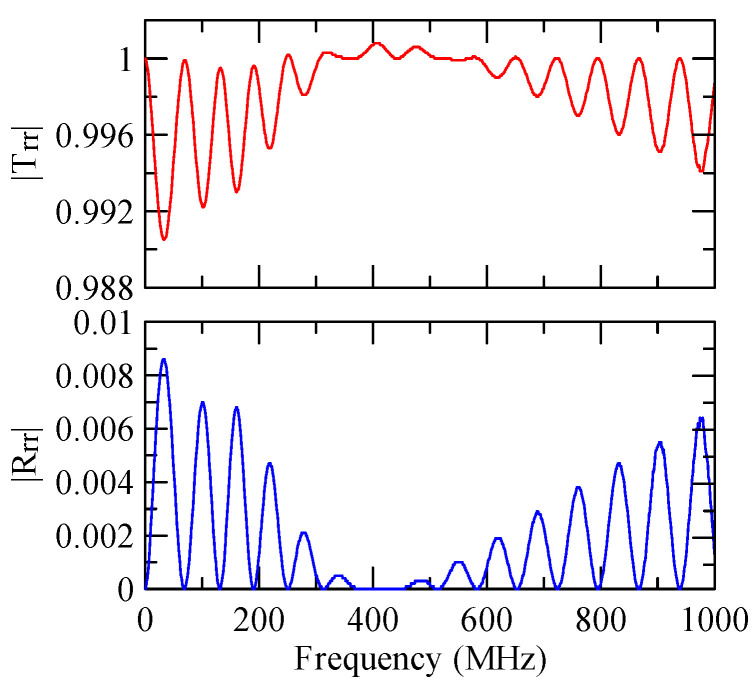
Reflection and transmission spectrum of $d_{a}=30\;\mu m$, $\Theta_{R}=45{}^{\circ}$, N = 1.

**Figure 16:**
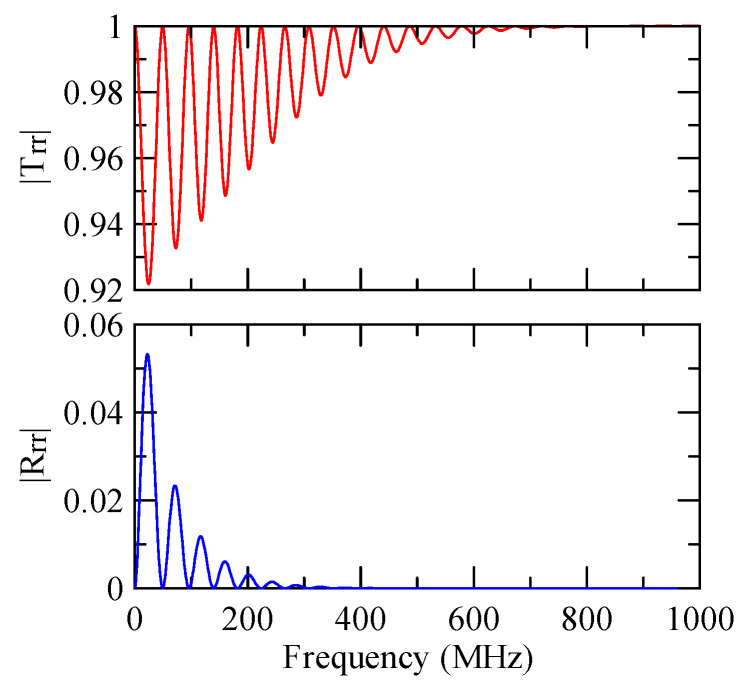
Reflection and transmission spectrum of $d_{a}=30\;\mu m$, $\Theta_{R}=0{}^{\circ}$, N = 1.

Figure 7 and Figure 8 show ZnO (1.5 μm)/glass and Al (0.5 μm)/ZnO (1.5 μm)/glass layered medium SAW phase velocity dispersion curves. According to the derivation in this section, the spectral changes in reflection and transmittance can be calculated when the Rayleigh wave is obliquely incident. When the Rayleigh wave passes through a single metal strip, the reflectivity and transmittance can be calculated according to the displacement of the layered medium’s upper surface. Figure 13, Figure 14, Figure 15 and Figure 16 show the reflection and transmission spectra corresponding to different strip widths and incident angles.

## 4. Simulation Results and Discussion

As a result of the derivation described in the previous section, this study utilizes the Al/ZnO/Glass array strip structure as an example, develops a FORTRAN numerical program, and calculates the effect of different strip arrays and design parameters on the reflection and transmission spectrum of SAW.

### 4.1. Frequency Analysis of Grating Arrays

#### 4.1.1. Effects of Strip Width and Incident Angle

**Figure 17:**
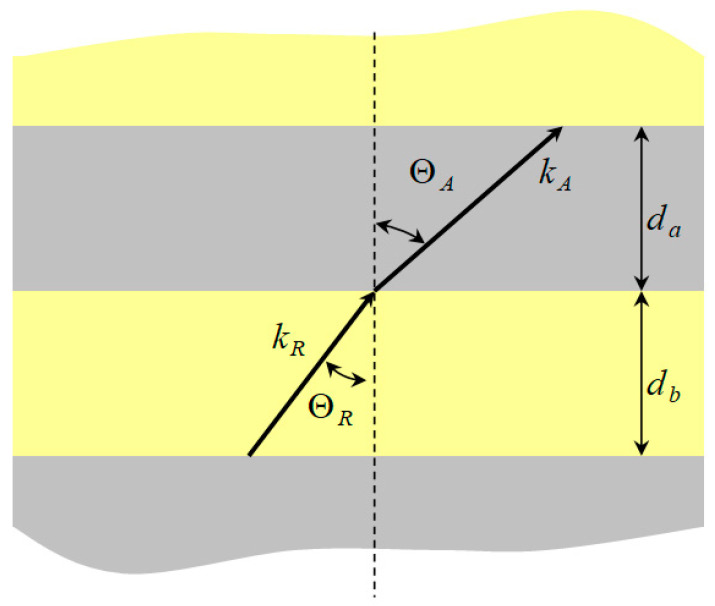
Diagram of wave propagation direction.

For analysis, the reflection and transmission spectra frequency axes are considered to be dimensionless. In Figure 17, the horizontal axis is represented as follows: $k_{A}d_{a}\cos\Theta_{A}/2\pi$. Here,

$k_{A}\cos\Theta_{A}$ is the component of the SAW wave number in the $x_{2}$ direction when the dispersion occurs. Subscript *A* represents the Rayleigh wave passing in the array strips.

**Figure 18:**
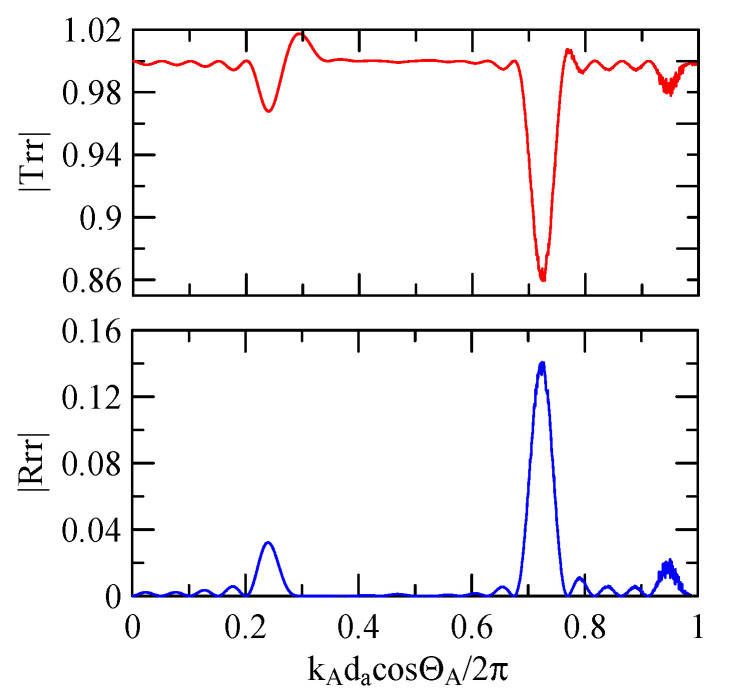
Reflection and transmission spectrum of $d_{a}=d_{b}=3\;\mu m$, $\Theta_{R}=45{}^{\circ}$, N = 5.

**Figure 19:**
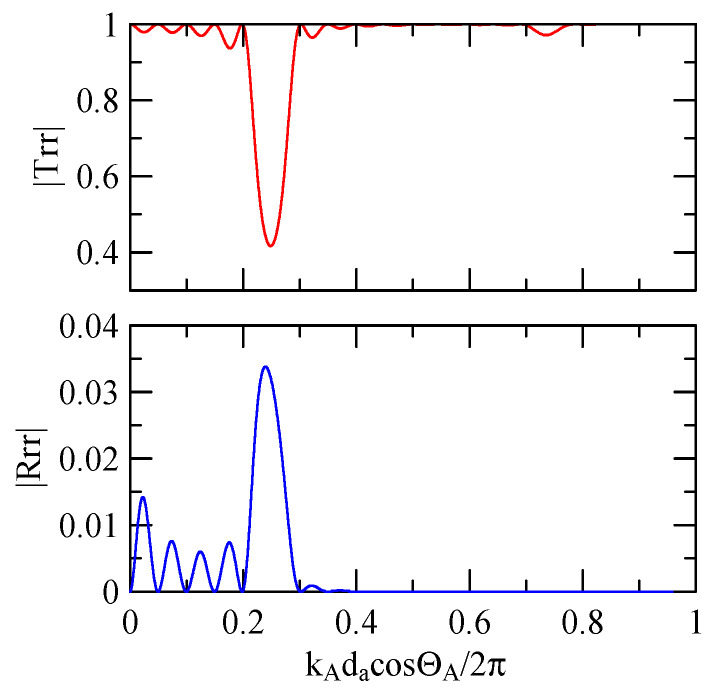
Reflection and transmission spectrum of $d_{a}=d_{b}=3\;\mu m$, $\Theta_{R}=0{}^{\circ}$, N = 5.

**Figure 20:**
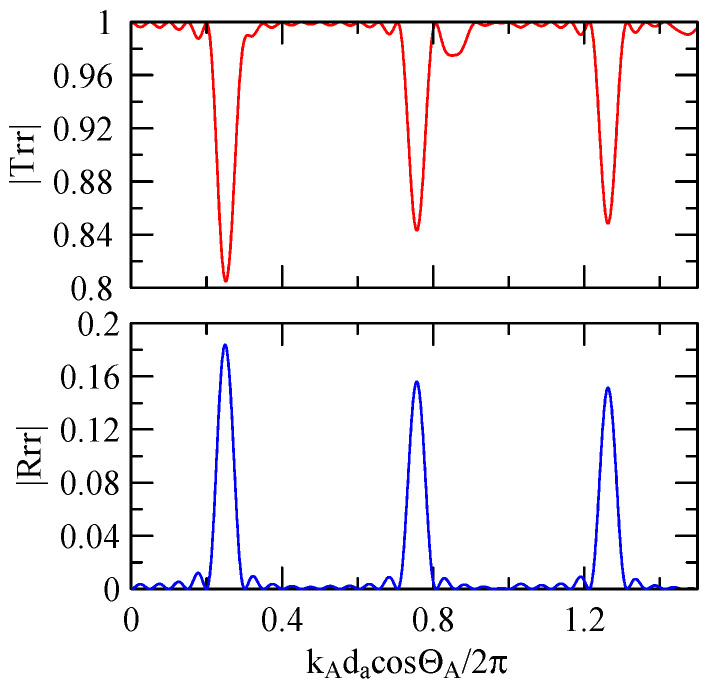
Reflection and transmission spectrum of $d_{a}=d_{b}=30\;\mu m$, $\Theta_{R}=45{}^{\circ}$, N = 5.

**Figure 21:**
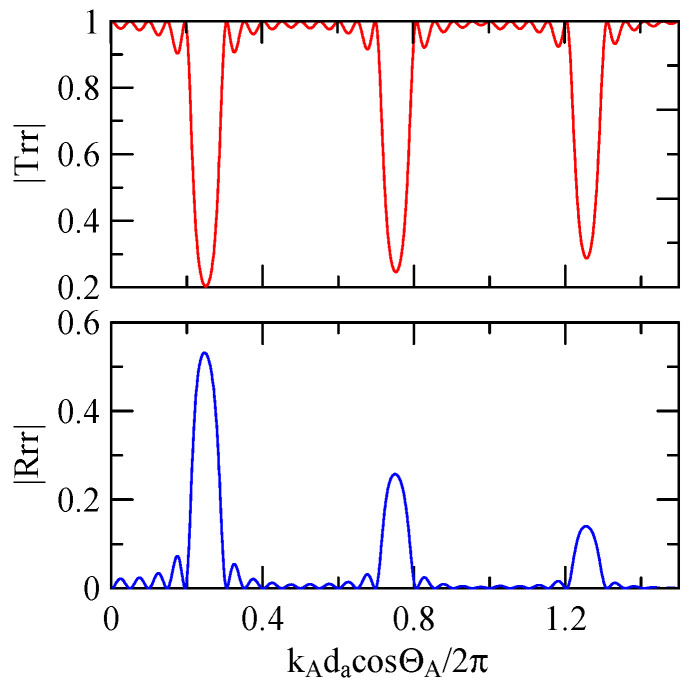
Reflection and transmission spectrum of $d_{a}=d_{b}=30\;\mu m$, $\Theta_{R}=0{}^{\circ}$, N = 5.

First consider the calculation example with a fixed number of strips, and the strip width and gap are equidistant. Figure 18, Figure 19, Figure 20 and Figure 21 show the spectrum diagrams of strip number N = 5, $d_{a}=d_{b}=3$, 30 μm. The incident angles are $\Theta_{R}=0{}^{\circ}$ and 45°. The figures show that the reflectivity and transmittance also differ when the strip widths or incident angles are different. However, at the abscissa $k_{A}d_{a}\cos\Theta_{A}/2\pi$ =0.5n+0.25(n∈N,n≥0), there is a local maximum (peak) reflectance and a local minimum (valley) transmittance. The frequency corresponding to this point is called the extreme frequency.

The SAW’s phase velocity in the strip area is different from the non-strip area. When a SAW is transmitted from a medium with a slower wave velocity to one with a faster wave velocity, the phase of the reflected wave generated at the interface will be delayed by one-half of a cycle. Therefore, the extreme frequency represents the wave transmission frequency producing constructive or destructive interferences.

**Figure 22:**
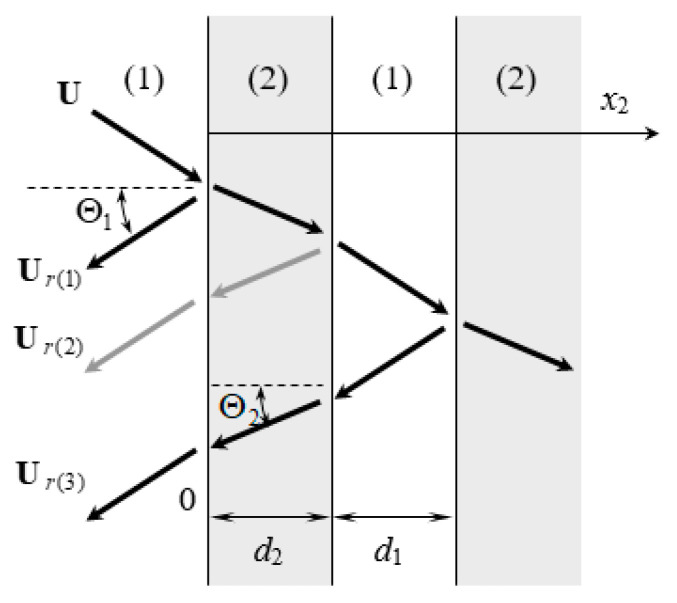
Diagram of reflected wave propagation.

In Figure 22, (1) and (2) represent different media. The media widths are $d_{1}$ and $d_{2}$, respectively, and U is the incident time-harmonic wave. $U_{r(i)}$ represents the first (or odd number) reflection wave propagation through the *i*-th interface, $\Theta_{1}$ and $\Theta_{2}$ are the reflection angles of SAWs in media (1) and (2), and $k_{1}$, $k_{2}$ are their wave numbers. If the wave velocity of medium (2) is slower than that of medium (1) and the wave propagation factor in the *x*_1_ direction is extracted, then:

(66) $$U_{r(1)}=\left|A_{1}\right|\exp(i\varepsilon_{0})\;U_{r(2)}=\left|A_{2}\right|\exp(i2k_{2}d_{2}\cos\Theta_{2}+i\varepsilon_{0}-i\pi )\;U_{r(3)}=\left|A_{3}\right|\exp(i2k_{2}d_{2}\cos\Theta_{2}+i2k_{1}d_{1}\cos\Theta_{1}+i\varepsilon_{0})\;k_{1}=2\pi /\lambda_{1},\;k_{2}=2\pi /\lambda_{2}$$

where $\left|A_{i}\right|$ and $\lambda_{i}$ correspond to the component and wavelength of the reflected wave amplitude of the *i*-th interface and $\varepsilon_{0}$ is the initial phase.

If $k_{1}d_{1}\cos\Theta_{1}\approx k_{2}d_{2}\cos\Theta_{2}$, when the ratio of the strip width to the wavelength component in the *x*_2_ direction is an odd multiple of 0.25, the reflected wave will have obvious constructive interference. The wave transmission and reflection number are even for the transmitted wave of the strip array, and its phase delay is exactly an integer period; therefore, there is no constructive interference. Destructive interference will only occur when the ratio of the strip width to wavelength component in the *x*_2_ direction is an odd multiple of 0.25.

#### 4.1.2. Effects of the Strip Number

**Figure 23:**
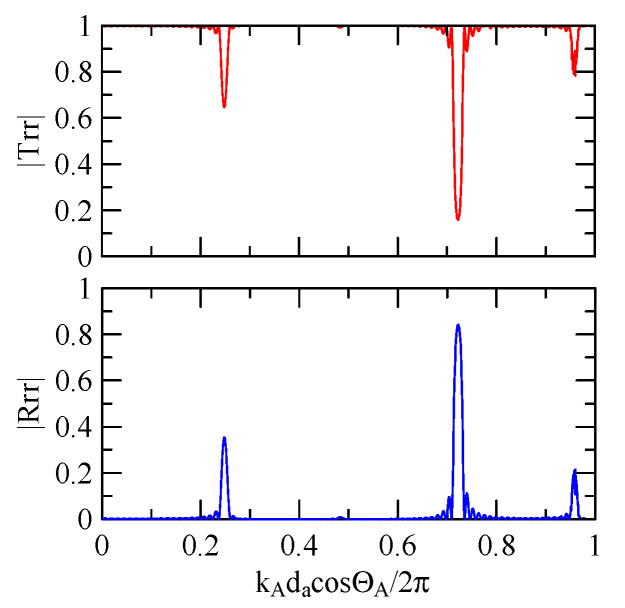
Reflection and transmission spectrum of $d_{a}=d_{b}=3\;\mu m$, $\Theta_{R}=45{}^{\circ}$, N = 20.

**Figure 24:**
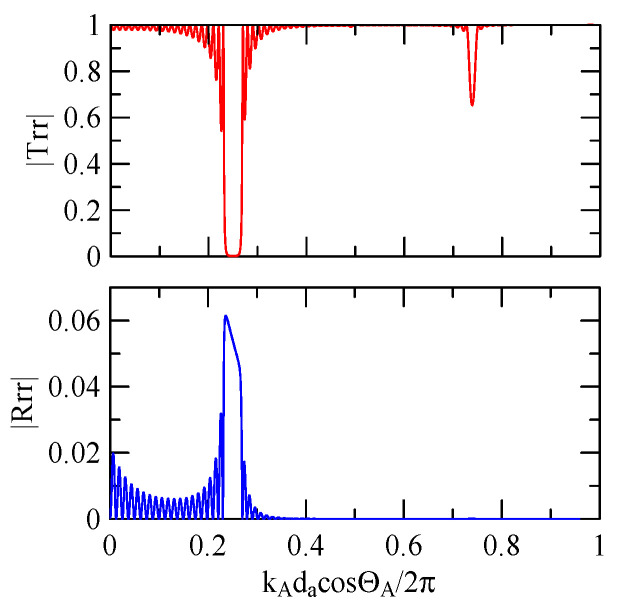
Reflection and transmission spectrum of $d_{a}=d_{b}=3\;\mu m$, $\Theta_{R}=0{}^{\circ}$, N = 20.

**Figure 25:**
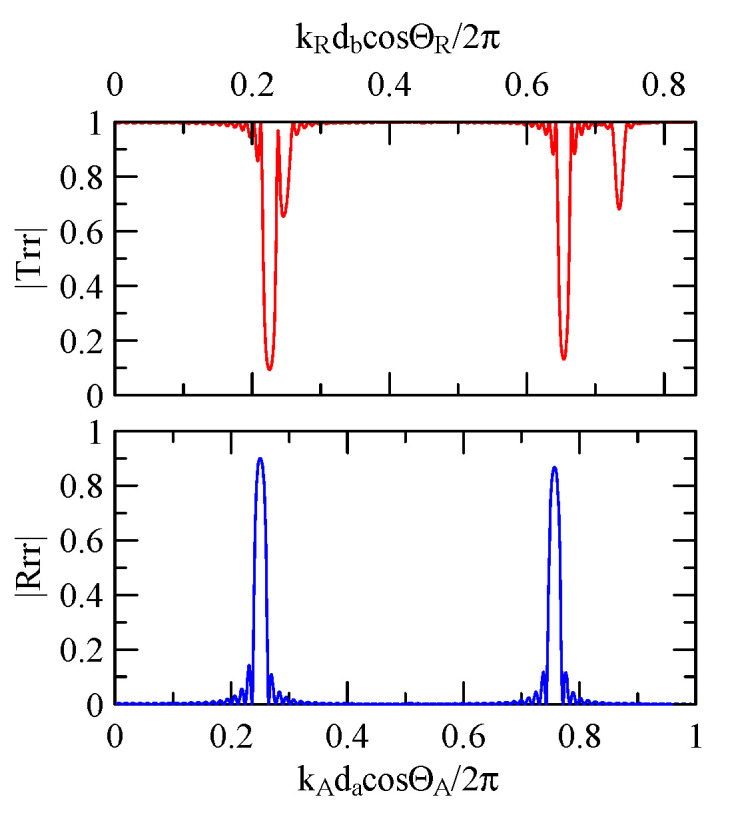
Reflection and transmission spectrum of $d_{a}=d_{b}=30\;\mu m$, $\Theta_{R}=45{}^{\circ}$, N = 20.

**Figure 26:**
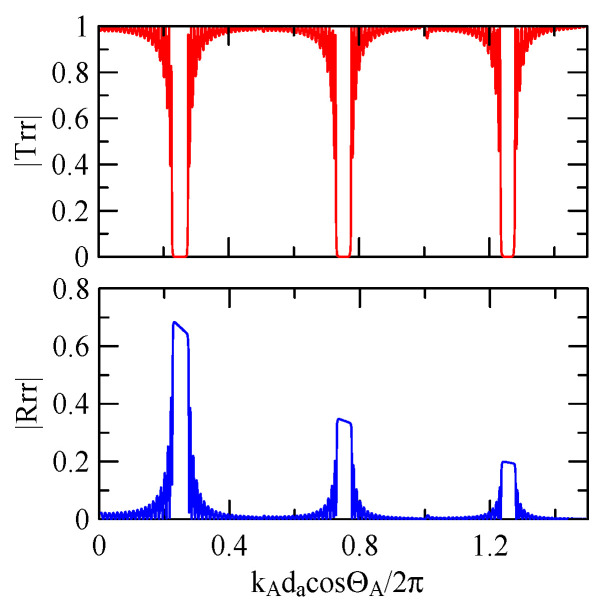
Reflection and transmission spectrum of $d_{a}=d_{b}=30\;\mu m$, $\Theta_{R}=0{}^{\circ}$, N = 20.

Figure 18, Figure 19, Figure 20 and Figure 21 show the reflection and transmission spectrums of the array strip number N = 5, and Figure 23, Figure 24, Figure 25 and Figure 26 display the reflection and transmission spectrums when the array strip number N = 20. Comparing the two shows that if the strip width and spacing are the same as the incident angle, the transmittance at the same frequency will decrease when the number of strips increases. Furthermore, the reflectance will increase accordingly, and the increase in strips will increase the reflectivity.

When the number of array strips increases, the SAW’s wave propagation is more obviously affected by the strip array boundary, and the construction or destructive interference caused by the strip width and gap is more significant. When the number of strips increases, the bandwidth of the extreme value of the reflectivity will be narrower. $k_{2(A)}d_{a}/2\pi$ =0.5n+0.25 is the extreme frequency.

#### 4.1.3. Effects of Strip Height

**Figure 27:**
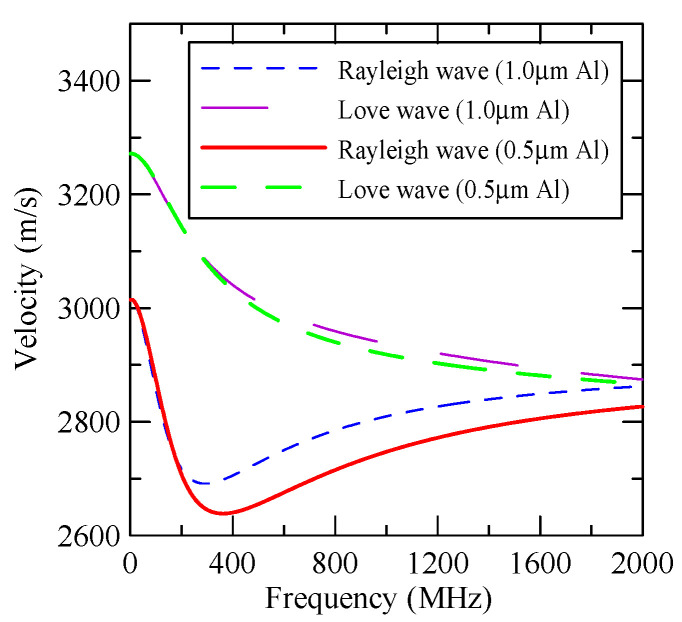
Dispersion curve of changes in the thickness of aluminum film.

**Figure 28:**
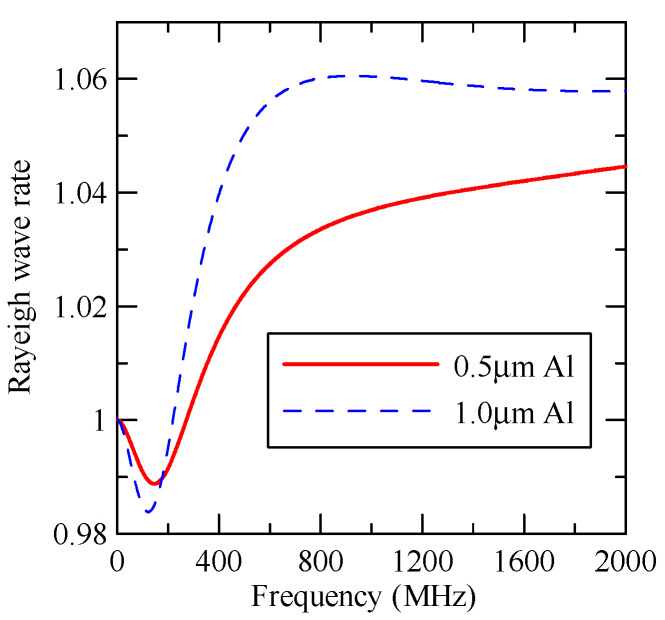
Comparison of Rayleigh wave phase velocities with different thicknesses of aluminum films.

Figure 27 shows the first mode Rayleigh and Love wave dispersion curves of Al/ZnO (1 μm)/glass layered medium with an aluminum film thickness of 0.5 and 1 μm. Figure 28 shows the Raleigh wave velocity ratio of the first mode of the covered aluminum film with a thickness of 0.5 and 1 μm and an uncovered ZnO (1 μm)/glass layered semi-infinite field medium. When the operating frequency is very low, the layered medium’s SAW phase velocity value is close to the SAW velocity of the substrate, regardless of the changes in the substrate’s coating medium. As the operating frequency increases, the coating medium gradually affects Rayleigh wave’s velocities or relative ratios.

**Figure 29:**
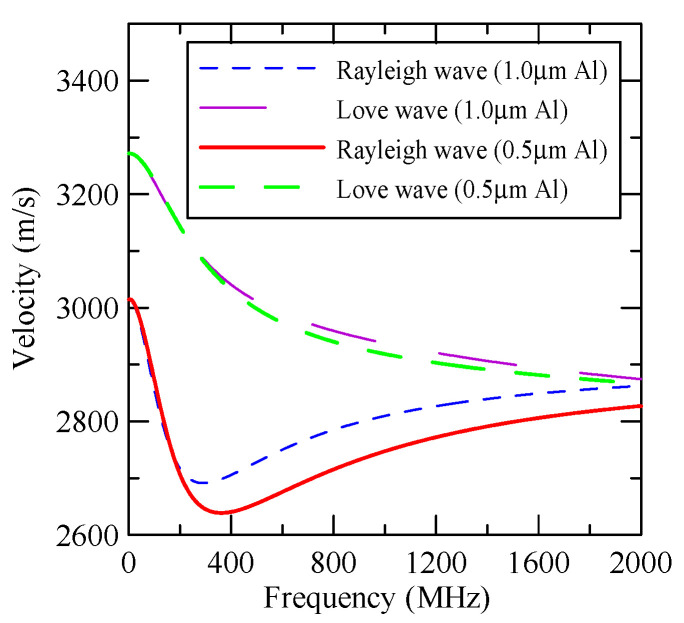
Reflection and transmission spectrum of $d_{a}=d_{b}=30\;\mu m$, $\Theta_{R}=45{}^{\circ}$, N = 20 (The thickness of Al is 1 μm).

**Figure 30:**
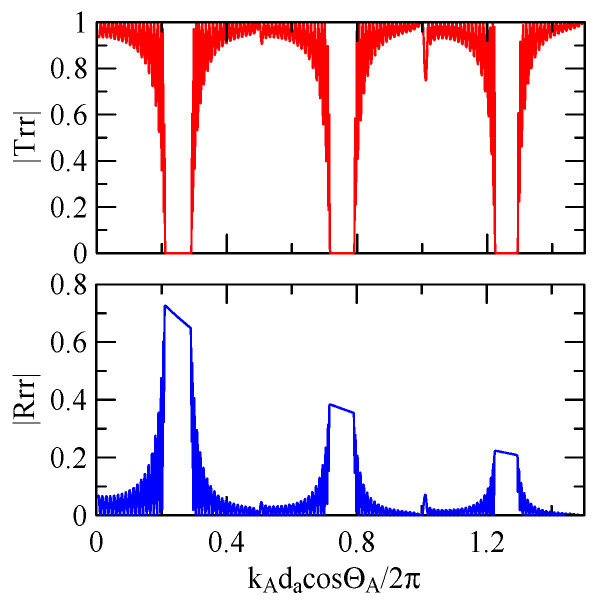
Reflection and transmission spectrum of $d_{a}=d_{b}=30\;\mu m$, $\Theta_{R}=0{}^{\circ}$, N = 20 (The thickness of Al is 1 μm).

Figure 29 and Figure 30 show the SAW’s reflection and transmission spectra incident on the 1 μm high aluminum strip array with $\Theta_{R}=45{}^{\circ}$ and 0°. These are compared with the spectrums of the 0.5 μm high aluminum strip array shown in Figure 25 and Figure 26. If the height of the strip increases, alongside the more evident reflection phenomenon, the bandwidth of the extreme frequency will also be wider. This is because the difference between the SAW phase velocity in the strip and the non-strip areas is more obvious; therefore, the extreme frequency is no longer limited to $k_{A}d_{a}\cos\Theta_{A}/2\pi =$ 0.5n+0.25.

#### 4.1.4. Unequal Gap and Strip Widths

When strip width and gap differ significantly, the extreme frequency of reflection and transmittance will shift. This shift depends on the dimensionless coordinates corresponding to the Rayleigh wave of the medium with strips or wider gap.

**Figure 31:**
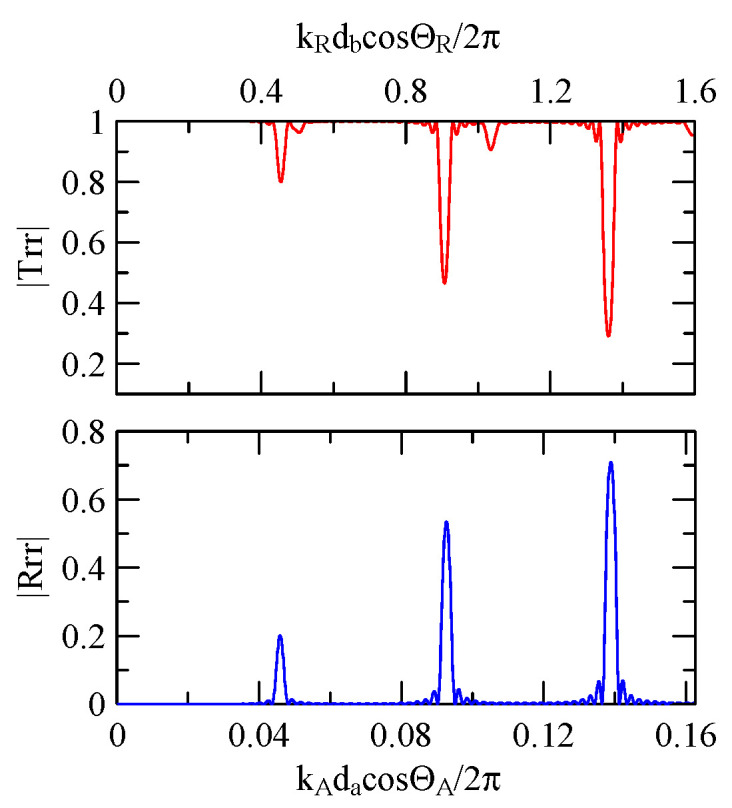
Reflection and transmission spectrum of $d_{a},d_{b}=3,30\;\mu m$, $\Theta_{R}=45{}^{\circ}$, N = 20.

**Figure 32:**
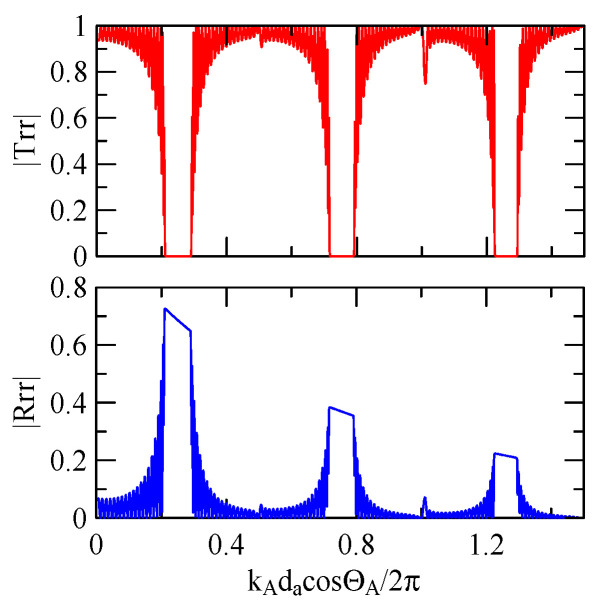
Reflection and transmission spectrum of $d_{a},d_{b}=30,3\;\mu m$, $\Theta_{R}=45{}^{\circ}$, N = 20.

**Figure 33:**
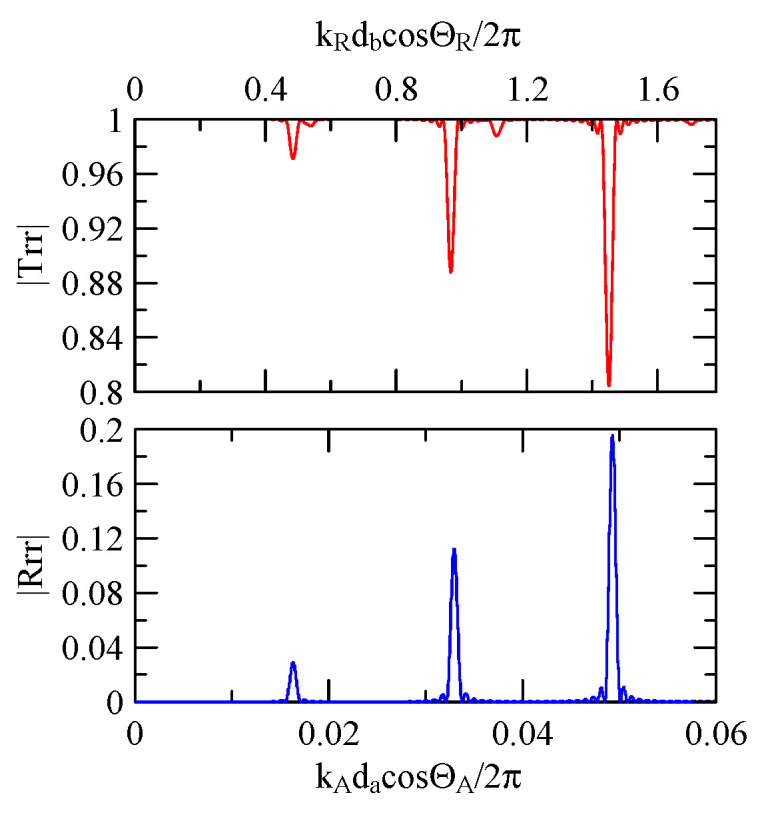
Reflection and transmission spectrum of $d_{a},d_{b}=1,30\;\mu m$, $\Theta_{R}=45{}^{\circ}$, N = 20.

**Figure 34:**
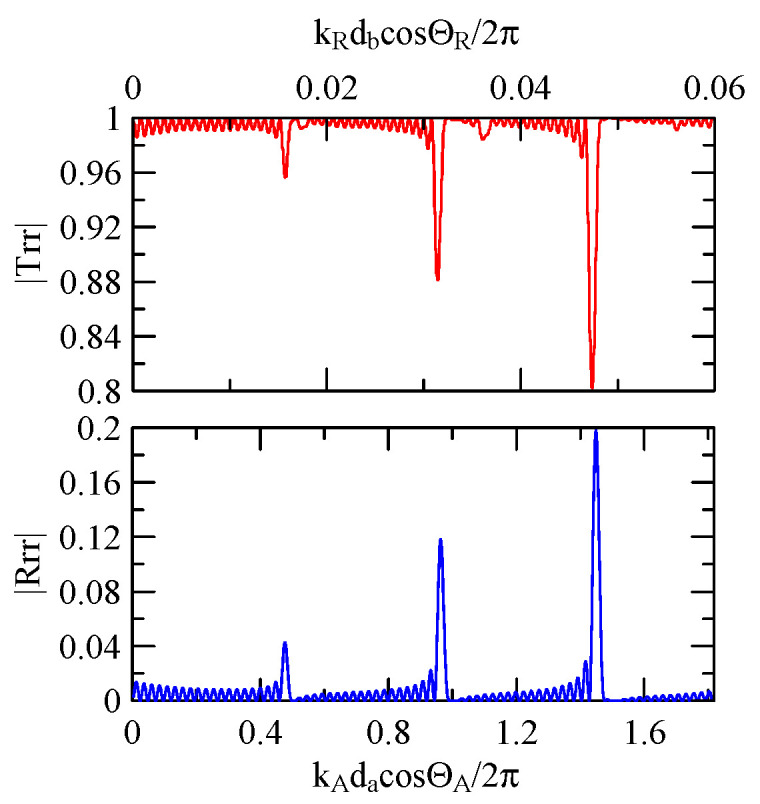
Reflection and transmission spectrum of $d_{a},d_{b}=30,1\;\mu m$, $\Theta_{R}=45{}^{\circ}$, N = 20.

**Figure 35:**
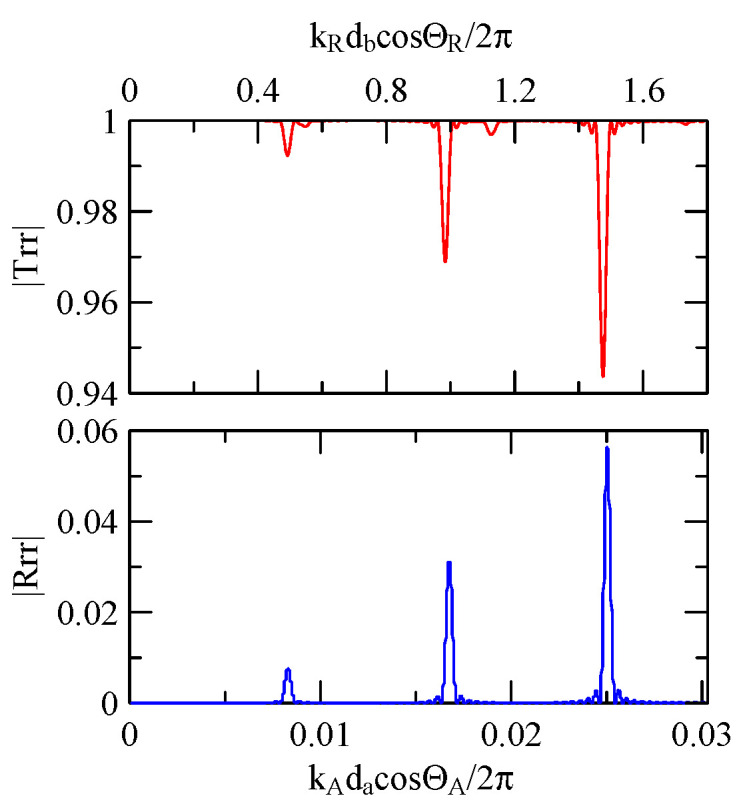
Reflection and transmission spectrum of $d_{a},d_{b}=0.5,30\;\mu m$, $\Theta_{R}=45{}^{\circ}$, N = 20.

**Figure 36:**
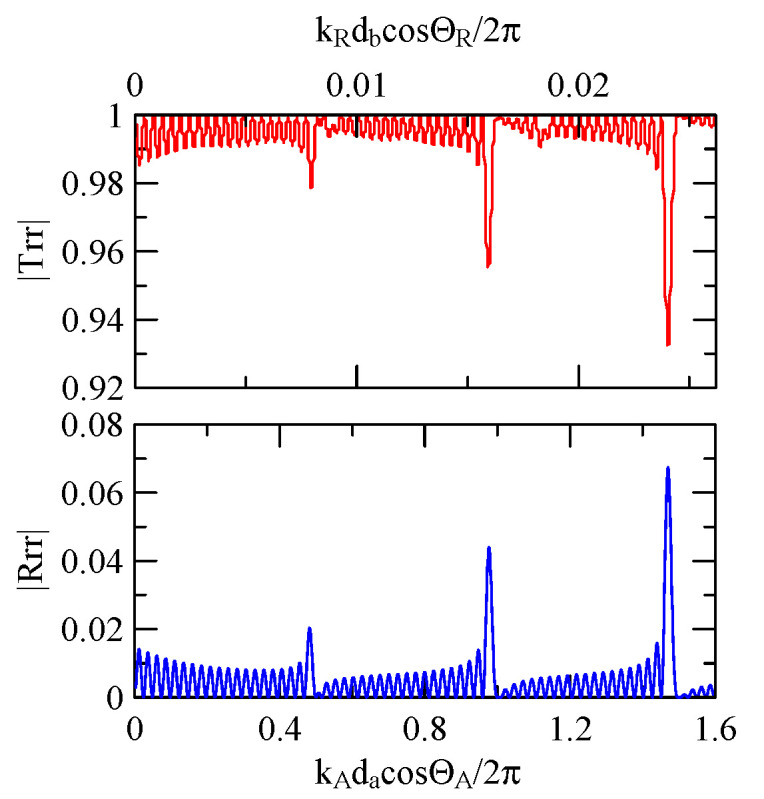
Reflection and transmission spectrum of $d_{a},\;d_{b}=30,0.5\;\mu m$, $\Theta_{R}=45{}^{\circ}$, N = 20.

Take fixing 20 strips as an example, as shown in Figure 31, Figure 32, Figure 33, Figure 34, Figure 35 and Figure 36. The reflectance spectrum takes $k_{A}d_{a}\cos\Theta_{A}/2\pi$ as its abscissa coordinate, and the transmittance spectrum takes $k_{R}d_{b}\cos\Theta_{R}/2\pi$ as its coordinate. $k_{R}\cos\Theta_{R}$ is the $x_{2}$ direction component of the Rayleigh wave number transmitted in the aluminum strip pitch, and $d_{b}$ is the strip pitch width.

When the strip width is much larger than the gap, the extreme frequency will approach $k_{A}d_{a}\cos\Theta_{A}/2\pi =0.5n$. Conversely, when the strip width is much smaller than the gap, its extreme frequency will approach $k_{R}d_{b}\cos\Theta_{R}/2\pi =0.5n$.

Equation (66) demonstrates that if $d_{2}<<d_{1}$, meaning that $k_{2}d_{2}\cos\Theta_{2}$ and phase delay are negligible, the reflected wave propagation’s constructive interference will occur at $k_{1}d_{1}\cos\Theta_{1}/2\pi$ =0.5n. This is consistent with this extreme frequency shift phenomenon.

### 4.2. Effect of Initial Conditions on Frequency Response

#### 4.2.1. Dispersion Effects and Reflectivity

If initial conditions, such as the incident angle or the substrate material, are changed, the SAW reflection and transmission frequency response will also be affected. This study has previously demonstrated that when $k_{1}d_{1}\cos\Theta_{1}\approx k_{2}d_{2}\cos\Theta_{2}$, the extreme frequency of reflection and transmittance will approach $k_{i}d_{i}\cos\Theta_{i}/2\pi =0.5n+0.25$. When there is a significant difference between $k_{1}d_{1}\cos\Theta_{1}$ and $k_{2}d_{2}\cos\Theta_{2}$, the extreme frequency will be biased towards $k_{i}d_{i}\cos\Theta_{i}/2\pi =0.5n$, where *i* has a larger ratio.

Due to the effect of dispersion, when the Rayleigh wave propagates in strip and non-strip regions, its wave number ratio will change according to frequency. Take the Rayleigh wave of 45° obliquely incident on a group of periodic strip arrays with a strip width of 30 μm and a number *N* = 20 as an example.

**Figure 37:**
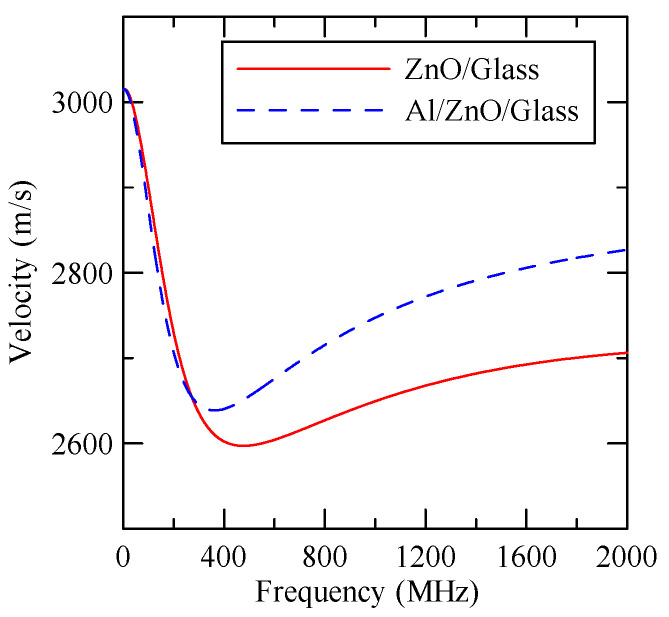
First mode Rayleigh wave dispersion curve of Al (0.5 μm)/ZnO (1.5 μm)/glass.

**Figure 38:**
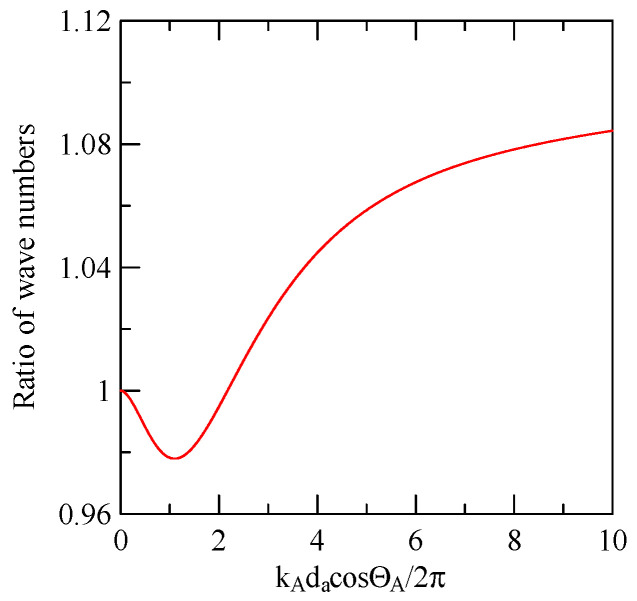
The wave number ratio of the $x_{2}$ direction between strip and non-strip areas.

**Figure 39:**
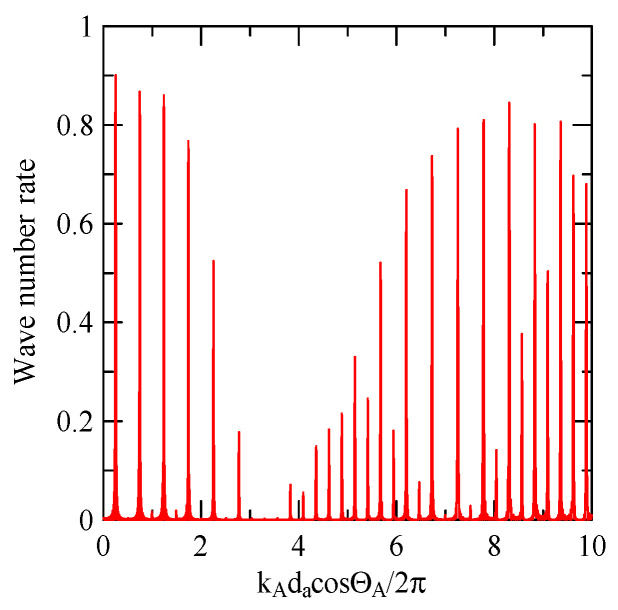
Reflectance spectrum of $d_{a}=d_{b}=30\;\mu m$, $\Theta_{R}=45{}^{\circ}$, N = 20.

Figure 37 shows the Rayleigh wave dispersion curve of the first mode of ZnO (1.5 μm)/glass structure and Al (0.5 μm)/ZnO (1.5 μm)/glass structure. Figure 38 displays the ratio curve of the wave number component in the $x_{2}$ direction between the strip and non-strip areas. Figure 39 shows its reflectivity spectrum diagram.

Comparing Figure 38 and Figure 39 demonstrates that when the strip width and gap are equal, but the wave numbers are slightly different, the reflectivity peak occurs at the original peak of $k_{A}d_{a}\cos\Theta_{A}/2\pi \approx 0.5n+0.25$ and at $k_{A}d_{a}\cos\Theta_{A}/2\pi$ ≈0.5n. However, the latter peak is less evident than the former.

#### 4.2.2. Oblique and Vertical Incidence

When the Rayleigh wave is obliquely incident, a mode conversion will occur at the interface between strips and non-strips and a Love wave will be generated. Therefore, in Figure 20 and Figure 25, the extreme transmittance frequency does not only appear at $k_{A}d_{a}\cos\Theta_{A}/2\pi =$0.5n+0.25(n∈N,n≥0). When the Rayleigh wave is vertically incident, there is no such phenomenon because there is no Love wave.

Taking Figure 25 as an example, the abscissa axis of the transmission spectrogram is changed to $k_{C}d_{a}\cos\Theta_{C}/2\pi$. When it is close to 0.5n+0.25(n∈N,n≥0), the lowest valley value also appears. $k_{C}\cos\Theta_{C}$ is the $x_{2}$ direction wave number of the Love wave in the strip.

#### 4.2.3. Global Notch of Reflectivity

**Figure 40:**
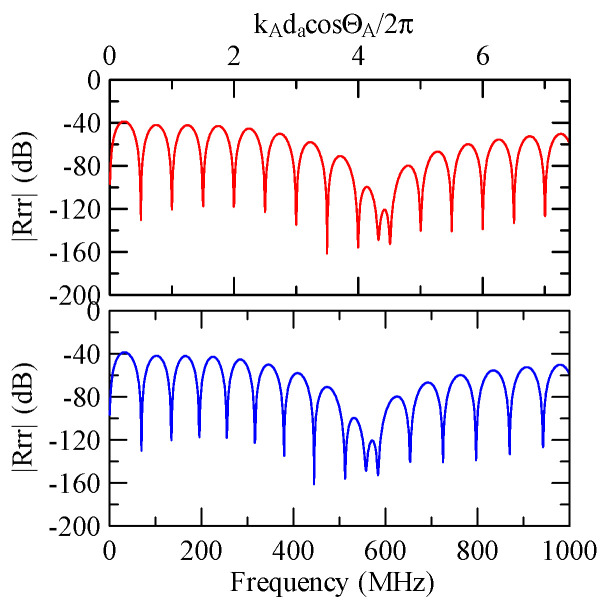
Reflectance response of $d_{a}=30\;\mu m$, $\Theta_{R}=45{}^{\circ}$, N = 1, and 1 μm thickness of Al.

**Figure 41:**
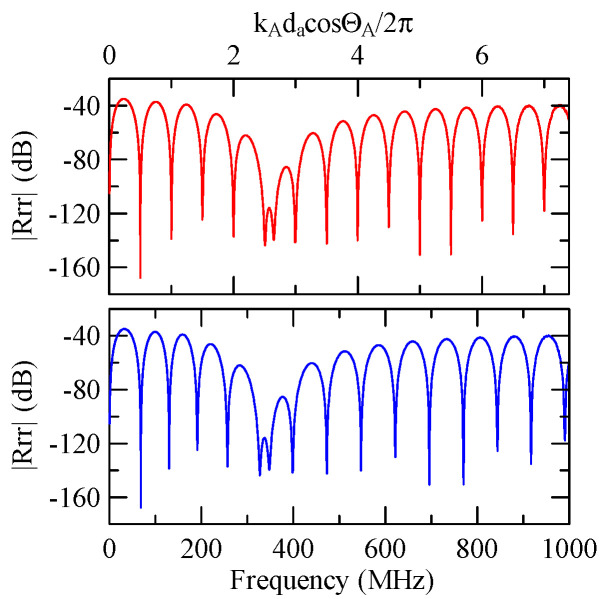
Reflectance response of $d_{a}=30\;\mu m$, $\Theta_{R}=45{}^{\circ}$, N = 1, and 1 μm thickness of Al.

**Figure 42:**
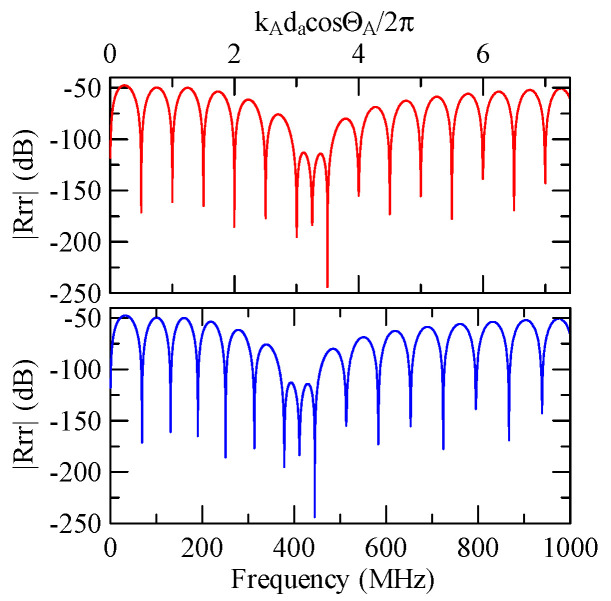
Reflectance response of $d_{a}=30\;\mu m$, $\Theta_{R}=45{}^{\circ}$, N = 1, and 1.5 μm thickness of ZnO.

**Figure 43:**
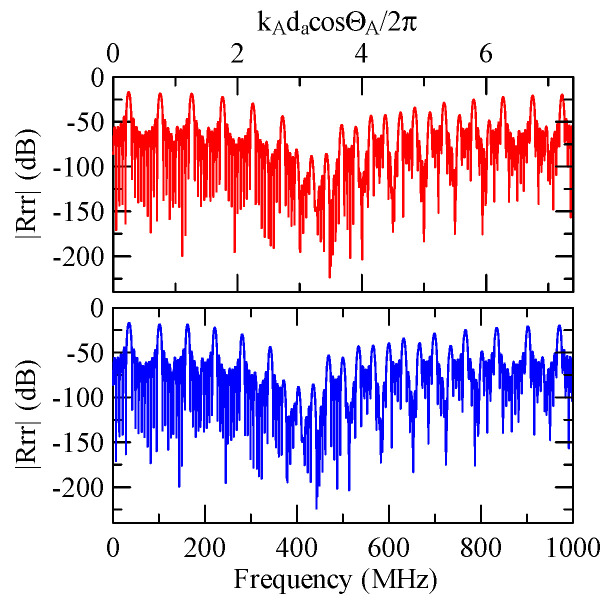
Reflectance response of $d_{a}=d_{b}=30\;\mu m$, $\Theta_{R}=45{}^{\circ}$, N = 5.

**Figure 44:**
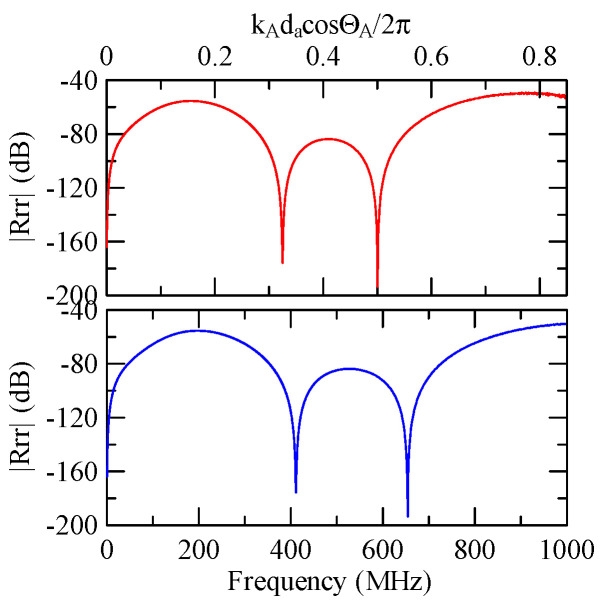
Reflectance response of $d_{a}=3\;\mu m$, $\Theta_{R}=45{}^{\circ}$, N = 1.

**Figure 45:**
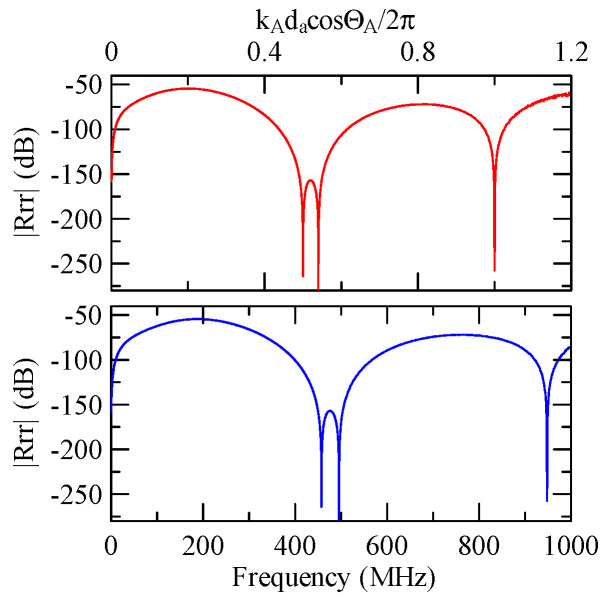
Reflectance response of $d_{a}=3\;\mu m$,$\Theta_{R}=15{}^{\circ}$, N = 1.

**Figure 46:**
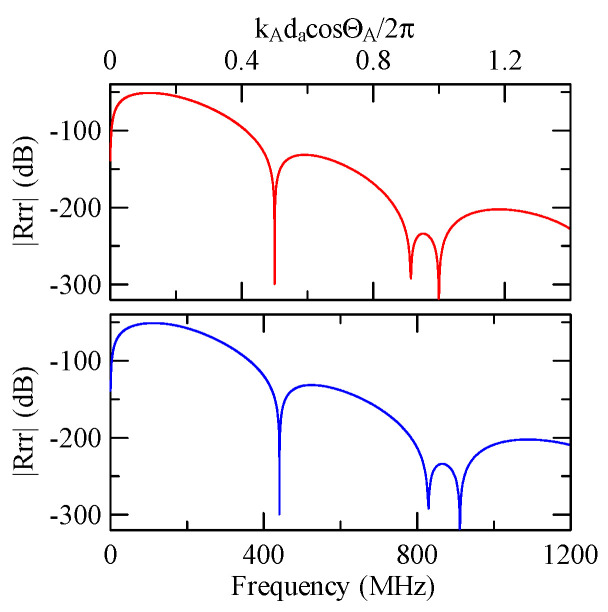
Reflectance response of $d_{a}=3\;\mu m$, $\Theta_{R}=0{}^{\circ}$, N = 1.

Assuming that Rayleigh waves are incident on an aluminum strip array of Al/ZnO/glass layered medium, Figure 40, Figure 41, Figure 42, Figure 43, Figure 44, Figure 45 and Figure 46 show the reflectivity responses obtained with different design parameters. To improve the resolution of smaller values in the figures, the reflectivity is changed to dB. From Figure 40 to Figure 42, the incident angle and strip array are the same, except that the thickness of the zinc oxide coating changes.

Figure 42, Figure 43 and Figure 44 show the simulation results of changing the number or width of the strip array. The thickness of the aluminum strips and zinc oxide are both 0.5 and 1 μm. The results shown in Figure 44, Figure 45 and Figure 46 highlight their different incident angles, and the other parameters are the same.

The above simulation results show that although different design parameters change the reflectivity and extreme frequency values, at the dimensionless coordinate $k_{A}d_{a}\cos\Theta_{A}/2\pi \approx 0.5n$, the reflected wave propagation is destructive interference. This is consistent with the previous section.

The global notch of reflectivity phenomenon can be identified around 560 MHz in Figure 40, 350 MHz in Figure 41, and 407 MHz in Figure 42, Figure 43 and Figure 44. From Figure 40 to Figure 42, alongside the different notch frequencies, the dispersion curve of the acoustic wave’s phase velocity on the surface of the layered medium also differs, representing a difference in material characteristics.

The layered media in Figure 42, Figure 43 and Figure 44 have the same dispersion curves, and the global notch occurs at the same frequency. As shown in Figure 44, Figure 45 and Figure 46, the notch frequency will change when the incident angle changes. When the incident angle is smaller, the frequency of the global notch tends to be higher.

Therefore, the frequency corresponding to the global notch phenomenon of reflectivity is only related to the medium’s material, the phase velocity of the SAW, and the wave propagation direction. This is unrelated to shape factors such as the strip array’s width, gap, and number.

### 4.3. Discussion

Several simulation methods are available for simulating surface acoustic wave (SAW) devices, each with their own advantages and limitations. Here is a comparison of some common SAW simulation methods:

1. Analytical method:

The analytical models provide simplified mathematical descriptions of SAW behavior, as well as quick estimations of device performance and behavior under ideal conditions [25].

Analytical models are, however, limited by their simplifications and assumptions and may not capture all the nuances of real-world SAW devices. They are appropriate exploratory tests of new designs.

2. Finite Element Method (FEM):

The FEM is a versatile numerical method that can accurately model complex geometrical features and material properties. The FEM can handle both 2D and 3D simulations. It is well suited for SAW devices that have irregular shapes or are layered. Several commercial simulation programs are available for simulating SAW devices, including COMSOL Multiphysics [27,29,31,32], ANSYS [23,26,30]. These software programs offer user-friendly interfaces and a wide range of capabilities for simulating SAW devices. They often include dedicated modules for simulating acoustics and piezoelectric.

However, setting up FEM simulations may require a significant number of computational resources and expertise. In addition, these software packages may require a license, which can be quite expensive. The specific capabilities and ease of use of each package may also differ.

3. Boundary Element Method (BEM):

In particular, the BEM is well suited to the modeling of SAW devices with free surfaces (e.g., liquid–solid interfaces), which can easily handle problems involving surface waves [20].

Despite this, the BEM may have limitations when dealing with complex 3D geometries or interior boundaries. It is also sensitive to the mesh quality.

4. Semi-analytical method:

For modeling SAW propagation in anisotropic materials, semi-analytical methods, such as the Stroh formalism, can be used to provide a right balance between accuracy and computational efficiency. The purpose in this study is to develop a numerical program in FORTRAN that calculates the impact of different strip arrays and design parameters on SAW reflection and transmission spectra.

However, semi-analytical methods may require specialized knowledge and code development. They are most applicable to specific materials and crystallographic orientations.

The choice of simulation method depends on the complexity of the SAW device, the accuracy required, available computational resources, and the user’s expertise. In practice, a combination of methods, such as analytical models for initial design and finite element simulations for detailed analysis, is often employed to achieve accurate results efficiently. Experimentally validated simulations are crucial for ensuring that the chosen simulation method accurately represents the SAW devices’ real-world behavior.

The Stroh formalism, named after the British mathematician and physicist William Prager and the American engineer Melvin Stroh [9], is a mathematical technique used to analyze the anisotropic materials’ elastic behavior. It is particularly useful for understanding the behavior of materials with complex crystalline structures, such as single crystals or certain types of composites.

It is important to note that Stroh formalism involves complex mathematical derivations and calculations. It is typically used by researchers and engineers with expertise in solid mechanics and materials science. It provides a powerful tool for understanding and predicting the behavior of anisotropic materials. This is especially true in cases where simpler models do not capture the complexity of the material’s response.

This study can be used as a reference for future SAW device designs. It can quantitatively simulate SAW’s wave propagation properties in strip arrays. It can also change the direction of reflection and transmission signals by adjusting the strip array’s angles or incident signals. Alongside picking up or blocking signals in a specific frequency range, a SAW device can have more diverse designs.

## 5. Conclusions

This study used the eighth-order piezoelectric wave equation to obtain the SAW’s phase velocity dispersion curve in layered media and each medium’s state vectors. The reflection and transmittance of SAWs propagating between surface strips was deduced by considering continuous wave propagation conditions between strip arrays. A numerical program was developed to simulate the influence of various metal strip array design parameters on reflection and transmittance.

The wave propagation of SAW in the strip array was affected by differences in the metal strip and substrate. Of these differences, the shape factor of the metal strip had the most significant influence. After simulation analysis, the following results were obtained:

1. When the width and gap of metal array strips are the same, the phase velocity difference in SAW in the strip and non-strip areas is insignificant when the reflectivity peak occurs at the dimensionless coordinate $k_{A}d_{a}\cos\Theta_{A}/2\pi$ =0.5n+0.25.
2. With an increase in the number of array strips, the reflectivity of SAWs will increase, which will narrow the bandwidth of the extreme frequency.
3. When the array strip height increases, alongside enhancing reflectivity, the difference between the SAW phase velocity in the strip area and the non-strip area becomes larger. As a result, the bandwidth of the extreme frequency increases.
4. When the difference between $k_{A}d_{a}\cos\Theta_{A}$ and $k_{R}d_{b}\cos\Theta_{R}$ increases due to differences in the gap and width of the strips or difference in wave velocity between the strip and non-strip area, the extreme frequency of the reflectivity will move. In general, this tends to occur near where the dimensionless coordinate of the one with the larger ratio of kdcosΘ is 0.5n.
5. The reflectivity spectrum of the metal array strips exhibits a global notch phenomenon influenced by the shape factor. It is a function of material, phase velocity of the SAW, and wave propagation direction that determines the frequency of the notch. It is not a function of strip array size, gap, and number.
6. When a Rayleigh wave is obliquely incident, a mode conversion will occur at the interface between strips and non-strips and a Love wave will be generated. The notch frequency will change when the incident angle changes. When the incident angle is smaller, the frequency of the global notch tends to be higher.

This study successfully deduced and simulated the strip array’s wave propagation properties. In the future, this method could be compared with empirical measurement data from a SAW device. It could also be extended to the simulation of the transducer, considering the attenuation of wave transmission energy. This would make SAW device simulation more complete.

## Figures and Tables

**Table 1:** Glass material constants.

Density (g/cm^3^)	Lame Constants (GPa)		Poisson Ratio	Dielectric Constant (10^−12^ F/m)
ρ	λ	μ	ν	$\varepsilon_{11}$
2.484	23.953	29.228	0.229	64.634

**Table 2:** Zinc oxide (ZnO) material constants.

Density (g/cm^3^)	Stiffness Coefficients (GPa)				
ρ	$C_{11}^{E}$	$C_{12}^{E}$	$C_{13}^{E}$	$C_{33}^{E}$	$C_{44}^{E}$
5.676	209.7	121.1	105.1	210.9	42.5
Piezoelectric Constants (C/m^2^)			Dielectric Constants (10^−12^ F/m)		
$e_{15}$	$e_{31}$	$e_{33}$	$\varepsilon_{11}^{S}$	$\varepsilon_{33}^{S}$	
−0.59	−0.61	1.14	73.8	78.3	

**Table 3:** Aluminum (Al) material constants.

Density (g/cm^3^)	Young’s Modulus (GPa)	Poisson Ratio	Dielectric Constants (10^−12^ F/m)
ρ	E	ν	$\varepsilon_{11}$
2.7	70	0.33	15.045

## Data Availability

Not Applicable.

## Appendix A

**Γ** is an 8×8 material property matrix:

(A1) $$\Gamma =\left[\begin{array}{cccc} \Gamma_{11} & \Gamma_{12} & \Gamma_{13} & \Gamma_{14}\\ \Gamma_{21} & \Gamma_{22} & \Gamma_{23} & \Gamma_{24}\\ \Gamma_{31} & \Gamma_{32} & \Gamma_{33} & \Gamma_{34}\\ \Gamma_{41} & \Gamma_{42} & \Gamma_{43} & \Gamma_{44} \end{array}\right]$$

Its components are as follows:

(A2) $$\Gamma_{11}=-Q*{\left(m_{3}\right)}^{-1}R*(m_{3},m_{\alpha })\partial_{\alpha }\;\Gamma_{12}=-iQ*{\left(m_{3}\right)}^{-1}\frac{S(m_{3})}{\varepsilon_{33}}\;\Gamma_{13}=-iQ*{\left(m_{3}\right)}^{-1}\;\Gamma_{14}=-Q*{\left(m_{3}\right)}^{-1}P*(m_{3},m_{\alpha })\partial_{\alpha }\;\Gamma_{21}=-i\left[\begin{array}{l} P*(m_{\beta },m_{\alpha })\\ -P*(m_{3},m_{\alpha })Q*{\left(m_{3}\right)}^{-1}R*(m_{3},m_{\beta }) \end{array}\right]\partial_{\alpha }\partial_{\beta }\;\Gamma_{22}=\frac{-1}{\varepsilon_{33}}\left[P*(m_{3},m_{\alpha })Q*{\left(m_{3}\right)}^{-1}S(m_{3})+\varepsilon_{3\alpha }\right]\partial_{\alpha }\;\Gamma_{23}=-P*(m_{3},m_{\alpha })Q*{\left(m_{3}\right)}^{-1}\partial_{\alpha }\;\Gamma_{24}=i\left[\begin{array}{l} P*(m_{3},m_{\alpha })Q*{\left(m_{3}\right)}^{-1}P*(m_{3},m_{\beta })\\ +\varepsilon_{\alpha \beta }-\frac{\varepsilon_{3\alpha }\varepsilon_{3\beta }}{\varepsilon_{33}} \end{array}\right]\partial_{\alpha }\partial_{\beta }\;\Gamma_{31}=i\left\{\rho \frac{\partial^{2}}{\partial t^{2}}+\left[\begin{array}{l} R*{\left(m_{3},m_{\alpha }\right)}^{T}Q*{\left(m_{3}\right)}^{-1}R*(m_{3},m_{\beta })\\ -R*(m_{\alpha },m_{\beta }) \end{array}\right]\partial_{\alpha }\partial_{\beta }\right\}\;\Gamma_{32}=\frac{1}{\varepsilon_{33}}\left[P(m_{\alpha },m_{3})-R*{\left(m_{3},m_{\alpha }\right)}^{T}Q*{\left(m_{3}\right)}^{-1}S(m_{3})\right]\partial_{\alpha }\;\Gamma_{32}=\frac{1}{\varepsilon_{33}}\left[P(m_{\alpha },m_{3})-R*{\left(m_{3},m_{\alpha }\right)}^{T}Q*{\left(m_{3}\right)}^{-1}S(m_{3})\right]\partial_{\alpha }\;\Gamma_{33}=-R*(m_{\alpha },m_{3})Q*{\left(m_{3}\right)}^{-1}\partial_{\alpha }\;\Gamma_{41}=\frac{1}{\varepsilon_{33}}\left[P(m_{\alpha },m_{3})-S(m_{3})Q*{(m_{3})}^{-1}R*(m_{3},m_{\alpha })\right]\partial_{\alpha }\;\Gamma_{42}=\frac{-i}{\varepsilon_{33}}\left[S(m_{3})Q*{\left(m_{3}\right)}^{-1}\frac{S(m_{3})}{\varepsilon_{33}}-1\right]\;\Gamma_{43}=-iS(m_{3})\frac{Q*{\left(m_{3}\right)}^{-1}}{\varepsilon_{33}}\;\Gamma_{44}=\frac{-1}{\varepsilon_{33}}\left[S(m_{3})Q*{\left(m_{3}\right)}^{-1}P*(m_{\alpha },m_{3})+\varepsilon_{3\alpha }\right]\partial_{\alpha }$$

where the tensors **Q**(**n**), R(a,b), and R*(a,b) and the vectors **S**(**n**), P(a,b), and P*(a,b) are defined as follows:

(A3) $$R(a,b)\sim C_{ijkl}b_{l}a_{j}\;P(a,b)\sim b_{k}e_{kij}a_{j}\;R*(a,b)=R(a,b)+\frac{P(a,m_{3})⊗P(b,m_{3})}{\varepsilon_{33}}\;P*(a,b)=P(a,b)-\frac{P(a,m_{3})\varepsilon_{3b}}{\varepsilon_{33}}\;Q*(n)=R*(n,n)\;S(n)=P(n,n)$$

Here, subscripts i,j,k,l=1…3, and α,β=1…2.

## Appendix B

Consider the problem of Rayleigh waves in the quasi-isotropic plane of a layered medium. Let $a_{0}$ represent the ratio of the Rayleigh wave displacement’s longitudinal component to the vertical component *u* in the *x*_3_ direction, $a_{1}$ the ratio of the surface electric displacement to the displacement component in the *x*_3_ direction, and $a_{2}$ the ratio of the surface potential to the displacement component in the *x*_3_ direction. The state vector composed of surface displacement and electric displacement can be expressed as:

(A4) $$\bar{U}(h_{N})={\left[a_{0}u\cos\theta ,a_{0}u\sin\theta ,u,a_{1}u\right]}^{T}$$

If the electrical boundary condition is open ${\bar{D}}_{3}(h_{\left(N\right)})=0$, meaning that $a_{1}=0$, the amplitude ratio can be obtained from the dispersion Equation (45) as

(A5) $$a_{0}={\left[\frac{G_{23}^{N}G_{31}^{N}-G_{21}^{N}G_{33}^{N}}{G_{21}^{N}G_{32}^{N}-G_{22}^{N}G_{31}^{N}}\csc\theta \right]}_{x_{3}^{\left(N\right)}=h_{N}},\;\theta \neq n\pi \;a_{0}={\left[\frac{G_{12}^{N}G_{23}^{N}-G_{22}^{N}G_{13}^{N}}{G_{11}^{N}G_{22}^{N}-G_{21}^{N}G_{12}^{N}}\sec\theta \right]}_{x_{3}^{\left(N\right)}=h_{N}},\;\theta \neq (n-0.5)\pi$$

If the surface of the layered medium is a free boundary ${\bar{\tau }}_{i}(h_{\left(N\right)})=0$, the ratio of potential to displacement can be obtained from the impedance relationship of Equation (28) as follows:

(A6) $$\bar{\phi }(h_{\left(N\right)})=a_{2}u\;a_{2}={\left[a_{0}(G_{41}^{N}\cos\theta +G_{42}^{N}\sin\theta )+G_{43}^{N}\right]}_{x_{3}^{\left(N\right)}=h_{N}}$$

Substituting the relationship between displacement and boundary conditions back into Equation (23), the specific coefficient vector can be obtained as follows:

(A7) $$\left[\begin{array}{c} C_{1}^{N}\\ C_{2}^{N} \end{array}\right]={\left[\begin{array}{cc} \Phi_{1}^{N}(h_{N}) & 0\\ 0 & \Phi_{2}^{N}(h_{N}) \end{array}\right]}^{-1}{\left[\begin{array}{cc} A_{1}^{N} & A_{2}^{N}\\ L_{1}^{N} & L_{2}^{N} \end{array}\right]}^{-1}\bar{Κ}u$$

Where

(A8) $$\bar{Κ}={\left[\begin{array}{cccccccc} a_{0}\cos\theta & a_{0}\sin\theta & 1 & 0 & 0 & 0 & 0 & a_{2} \end{array}\right]}^{T}$$

Through iterative processing, the relationship between the state vector and specific coefficients of each layer in the layered medium can be obtained:

(A9) $$\left\{\begin{array}{c} \bar{U}(x_{3})\\ \bar{T}(x_{3}) \end{array}\right\}=\left[\begin{array}{cc} A_{1}^{m} & A_{2}^{m}\\ L_{1}^{m} & L_{2}^{m} \end{array}\right]\left[\begin{array}{cc} \Phi_{1}^{m}(x_{3}^{\left(m\right)}) & 0\\ 0 & \Phi_{2}^{m}(x_{3}^{\left(m\right)}) \end{array}\right]\left\{\begin{array}{c} C_{1}^{m}\\ C_{2}^{m} \end{array}\right\}$$

(A10) $$\left[\begin{array}{c} C_{1}^{m}\\ C_{2}^{m} \end{array}\right]={\left[\begin{array}{cc} A_{1}^{m} & A_{2}^{m}\\ L_{1}^{m} & L_{2}^{m} \end{array}\right]}^{-1}\sum_{m}^{N}\left(\left[\begin{array}{cc} A_{1}^{m} & A_{2}^{m}\\ L_{1}^{m} & L_{2}^{m} \end{array}\right]\Pi^{m}\right)\bar{Κ}u$$

where the matrix Π is related to the material properties of each layer as follows:

(A11) $$\Pi^{m}={\left[\begin{array}{cc} \Phi_{1}^{m}(h_{m}) & 0\\ 0 & \Phi_{2}^{m}(h_{m}) \end{array}\right]}^{-1}{\left[\begin{array}{cc} A_{1}^{m} & A_{2}^{m}\\ L_{1}^{m} & L_{2}^{m} \end{array}\right]}^{-1}$$

**If** the electrical boundary condition is closed $\bar{\phi }(h_{\left(N\right)})=0$, the state vector $\bar{Κ}$ is:

(A12) $$\bar{Κ}={\left[\begin{array}{cccccccc} a_{0}\cos\theta & a_{0}\sin\theta & 1 & a_{1} & 0 & 0 & 0 & a_{2} \end{array}\right]}^{T}$$

According to the Equation (28)’s dispersion relationship, each proportional coefficient can be derived as follows:

(A13) $$a_{0}={\left[G{’}_{21}^{N}\csc\theta \right]}_{x_{3}^{\left(N\right)}=h_{N}},\;\theta \neq n\pi \;a_{0}={\left[G{’}_{11}^{N}\sec\theta \right]}_{x_{3}^{\left(N\right)}=h_{N}},\;\theta \neq (n-0.5)\pi \;a_{1}={\left(G{’}_{31}^{N}\right)}_{x_{3}^{\left(N\right)}=h_{N}}\;a_{2}={\left[a_{0}(G_{41}^{N}\cos\theta +G_{42}^{N}\sin\theta )+G_{43}^{N}+a_{1}G_{44}^{N}\right]}_{x_{3}^{\left(N\right)}=h_{N}}$$

where $G{’}^{N}$ is found in Equation (29).

When the wave propagates as a transversely polarized Love wave, the state vector composed of the surface displacement and the electric displacement of the particle can be expressed as follows:

(A14) $$\bar{U}(h_{\left(N\right)})={\left[u\sin\theta ,-u\cos\theta ,0,b_{0}u\right]}^{T}$$

where u is the Love wave’s displacement in the transverse polarization of the surface.

If the electrical boundary condition is open ${\bar{D}}_{3}(h_{\left(N\right)})=0$ and the surface of the layered medium is a free boundary ${\bar{\tau }}_{i}(h_{\left(N\right)})=0$, the ratio of potential to displacement can be obtained from the impedance relationship in Equation (28) as follows:

(A15) $$\bar{U}(h_{\left(N\right)})={\left[u\sin\theta ,-u\cos\theta ,0,b_{0}u\right]}^{T}$$

(A16) $$\bar{\phi }(h_{\left(N\right)})=b_{1}u\;b_{1}={\left(G_{41}^{N}\sin\theta -G_{42}^{N}\cos\theta \right)}_{x_{3}^{\left(N\right)}=h_{N}}$$

Substituting back into Equation (23) and extending to the N-layer structure, the specific coefficient vector can be expressed as follows:

(A17) $$\left[\begin{array}{c} C_{1}^{m}\\ C_{2}^{m} \end{array}\right]={\left[\begin{array}{cc} A_{1}^{m} & A_{2}^{m}\\ L_{1}^{m} & L_{2}^{m} \end{array}\right]}^{-1}\sum_{m}^{N}\left(\left[\begin{array}{cc} A_{1}^{m} & A_{2}^{m}\\ L_{1}^{m} & L_{2}^{m} \end{array}\right]\Pi^{m}\right)\bar{Κ}u$$

(A18) $$\Pi^{m}={\left[\begin{array}{cc} \Phi_{1}^{m}(h_{m}) & 0\\ 0 & \Phi_{2}^{m}(h_{m}) \end{array}\right]}^{-1}{\left[\begin{array}{cc} A_{1}^{m} & A_{2}^{m}\\ L_{1}^{m} & L_{2}^{m} \end{array}\right]}^{-1}$$

Where

(A19) $$\bar{Κ}={\left[\begin{array}{cccccccc} \sin\theta & -\cos\theta & 0 & 0 & 0 & 0 & 0 & b_{1} \end{array}\right]}^{T}$$

When the electrical boundary condition is closed $\bar{\phi }(h_{\left(N\right)})=0$, the state vector $\bar{Κ}$ can be written as follows:

(A20) $$\bar{Κ}={\left[{\begin{array}{cccccccc} \sin\theta & -\cos\theta & 0 & b_{0} & 0 & 0 & 0 & b \end{array}}_{1}\right]}^{T}$$

Similarly, the values of $b_{0}$ and $b_{1}$ can be calculated from Equations (47) and (28) as follows:

(A21) $$b_{0}={\left(\frac{G_{11}^{N}G_{42}^{N}-G_{41}^{N}G_{12}^{N}}{G_{12}^{N}G_{44}^{N}-G_{42}^{N}G_{14}^{N}}\sin\theta \right)}_{x_{3}^{\left(N\right)}=h_{N}},\;(\theta \neq n\pi )\;b_{0}={\left(\frac{G_{21}^{N}G_{44}^{N}-G_{41}^{N}G_{24}^{N}}{G_{12}^{N}G_{44}^{N}-G_{42}^{N}G_{14}^{N}}\cos\theta \right)}_{x_{3}^{\left(N\right)}=h_{N}},\;(\theta \neq (n-0.5)\pi )\;b_{1}={\left(G_{41}^{N}\sin\theta -G_{42}^{N}\cos\theta +b_{0}G_{44}^{N}\right)}_{x_{3}^{\left(N\right)}=h_{N}}$$
